# Benzoic acid facilitates ANF in monocot crops by recruiting nitrogen-fixing *Paraburkholderia*

**DOI:** 10.1093/ismejo/wrae210

**Published:** 2024-10-22

**Authors:** Ran Liu, Ruirui Li, Yanjun Li, Mingjia Li, Wenjing Ma, Lei Zheng, Cunhu Wang, Kefei Zhang, Ya Tong, Guoqiang Huang, Xinxin Li, Xin-Guang Zhu, Chuihuai You, Yongjia Zhong, Hong Liao

**Affiliations:** Root Biology Center, Fujian Agriculture and Forestry University, Fuzhou 350002, China; Root Biology Center, Fujian Agriculture and Forestry University, Fuzhou 350002, China; Root Biology Center, Fujian Agriculture and Forestry University, Fuzhou 350002, China; Root Biology Center, Fujian Agriculture and Forestry University, Fuzhou 350002, China; Root Biology Center, Fujian Agriculture and Forestry University, Fuzhou 350002, China; Root Biology Center, Fujian Agriculture and Forestry University, Fuzhou 350002, China; Root Biology Center, Fujian Agriculture and Forestry University, Fuzhou 350002, China; Root Biology Center, Fujian Agriculture and Forestry University, Fuzhou 350002, China; Root Biology Center, Fujian Agriculture and Forestry University, Fuzhou 350002, China; National Engineering Research Center of Sugarcane, Fujian Agriculture and Forestry University, Fuzhou, Fujian 350002, China; Root Biology Center, Fujian Agriculture and Forestry University, Fuzhou 350002, China; National Key Laboratory for Plant Molecular Genetics, CAS Center for Excellence in Molecular Plant Sciences, Shanghai Institute of Plant Physiology and Ecology, Chinese Academy of Sciences, Shanghai 200031, China; College of Life Sciences, Fujian Agriculture and Forestry University, Fuzhou 350002, China; Root Biology Center, Fujian Agriculture and Forestry University, Fuzhou 350002, China; Root Biology Center, Fujian Agriculture and Forestry University, Fuzhou 350002, China

**Keywords:** root microbiota, nitrogen, *paraburkholderia*, benzoic acid, sugarcane

## Abstract

Associative nitrogen fixation contributes large portion of N input to agro-ecosystems through monocot–diazotrophic associations. However, the contribution of associative nitrogen fixation is usually neglected in modern agriculture, and the underlying mechanisms of association between monocot and diazotrophs remain elusive. Here, we demonstrated that monocot crops employ mucilage and associated benzoic acid to specially enrich diazotrophic partners in response to nitrogen deficiency, which could be used for enhancing associative nitrogen fixation in monocot crops. To be specific, mucilage and benzoic acid induced in sugarcane roots by nitrogen deficiency mediated enrichment of nitrogen-fixing *Paraburkholderia* through specific recruitment whereas other bacteria were simultaneously repelled. Further studies suggest maize employs a similar strategy in promoting associations with diazotrophs. In addition, our results also suggest that benzoic acid application significantly increases copy numbers of the *nifH* gene in soils and enhances associative nitrogen fixation in maize using ^15^N enrichment assay. Taken together, these results reveal a mechanism regulating the association between monocot crops and nitrogen-fixing bacteria, and, thereby point towards ways to harness these beneficial microbes in efforts to increase nitrogen efficiency in monocot crops through pathways regulated by a specific signaling molecule.

## Introduction

Biological nitrogen fixation (BNF) is an essential component of nitrogen (N) cycling on earth [1]. It contributes about half of the total N input into agro-ecosystems [2, 3]. After the identification of BNF in the late 19th century, it has drawn great interest [4]. According to the associations between N-fixing microorganisms and host plants, BNF can be divided into symbiotic NF (SNF), associative NF (ANF), and free-living NF (FNF) [5–7]. Most studies have focused on SNF for its high efficiency of NF, and the symbiotic association between legumes and rhizobia has become a well-documented model of plant-dizotrophic bacteria association [6, 8]. Much less is known about ANF in nonlegume crops, especially in *Poaceae*.

*Poaceae* such as cereal crops are major staple crops and sources of food for humans and livestock [7]. Unlike SNF in legumes, ANF does not form obvious specialized structures, and it occurs in a wider range of plant species [6, 7, 9]. In this association, N-fixing bacteria colonize the rhizosphere, as well as intracellular and intercellular root tissues [7, 10, 11]. It is believed that the NF efficiency of ANF is lower than that BNF [9], which might be an explanation for mostly neglecting potential ANF capacity as about half of total industrial N production has been applied to major monocot crops, including maize, rice, and wheat [12]. Accounting for heedlessly neglected ANF contributions to NF and N input adds up to 50–70 Tg N per year to manage and possibly utilize across global agro-ecosystems [13, 14]. Abundant N fertilizer application may not only inhibit ANF capacity in monocot crop systems, but also has caused serious environmental problems [15–18]. Increasing our understanding of ANF regulation under various N status conditions and incorporating this knowledge into nutrient management plans, therefore, is necessary for sustainable monocot crop production.

ANF was first discovered in the C_4_ crop sugarcane in Brazil where it contributed up to 70.0% of the total N for growth and development [19]. ANF has been successfully used to supply N in Brazilian sugarcane fields receiving low N (LN) fertilizer inputs [20]. In addition, ANF also now knows to occur in other crops, such as maize [12, 21–23], sorghum [24], and rice [25, 26]. A recent study of an indigenous maize landrace accession revealed an association with N-fixing bacteria that suppled 28%–83.0% of the N acquired by host maize plants through N_2_ fixation facilitated by secretions of large amounts of mucilage from adventitious roots [27]. Therefore, understanding the association between monocot crops and diazotrophic microorganisms in the context to N status appears to be a promising avenue to explore for improvements in ANF across staple crops.

In this study, we first determined that LN availability drives specific enrichment of N-fixing *Paraburkholderia* in root systems of sugarcane adapted to N deficiency conditions. We then demonstrated that mucilage and associated BA attracted *Paraburkholderia* whereas repelling other bacteria, leading to specific enrichment of this N fixer in sugarcane rhizospheres. Similar selective enrichment was also discovered in maize, suggesting a potential general strategy among monocot crops. Our results present a model for how the host plant might select beneficial microbes through specific metabolites reflective of nutrient status. It also provides a strategy to manipulate plant associated microbiomes to increase nutrient acquisition.

## Materials and methods

### Plant material and cultivation

Sugarcane cultivar ROC22, which is widely cultivated in South China [28], was selected for this study. Maize cultivar MinTian6855, as provided by Yang Zhang of the Fujian Academy of Agriculture Science, and which is widely cultivated in Fujian Province, was used in experiments involving maize. For the HN and LN treatments of sugarcane plants, a field trial was carried out at the sugarcane experimental field station of Fujian Agriculture and Forestry University. Sugarcane plants were cultivated in a split plot design with of 0.8 m between plants in 15-m long plot rows spaced 50 cm apart. The basic physical and chemical soil properties are listed in Extended Data Table 2. For the high N (HN) treatment, 750 kg/ha of urea was applied to the field, and 225 kg/ha of urea was applied as the LN treatment. HN and LN plots were planted in four replicates.

Tissue culture seedlings of sugarcane were provided by the National Engineering Research Center of Sugarcane, Fujian Agriculture and Forestry University. After three days of hardening, seedlings were cultured in modified Hoagland's nutrient solution [29] ventilated for 15 min every hour. The hydroponic culture solution was renewed every three days.

For indoor pot culture assays, sugarcane seedlings were transplanted at the four-leaf stage to soil collected from the sugarcane field described above mixed with sterile perlite (121°C, 20 min) in a 1:1 ratio. Seedlings (one seedling per pot) were grown in pots (10 × 10 × 15) with one plant per pot. For LN and HN treatments, 100 ml of LN (500 μM) or HN nutrient solution (5000 μM) were supplied every three days. Maize plants were also included in the pot experiment, with four maize seeds sown per pot, which was thinned to one plant per pot seven days after germination. Maize seedlings were cultivated and treated as described above for sugarcane. For each treatment, 16 pots were prepared. Plants were placed in a growth chamber (day/night: 14 h/10 h, 26°C/24°C) under 37.5 μEm^−2^ s^−1^ of daytime light intensity for 40 days until samples were harvested.

### Sample collection for 16S rRNA sequencing

Field sampling was conducted by harvesting sugarcane plants at the elongation stage. Plants were dug out and roots within the depth range of 0–30 cm were collected by removing the bulk soil through shaking and washing with sterile PBS solution [30]. For each biological replicate, root samples were collected from five independent sugarcane plants in each plot and immediately moved to dry ice for storage until further treatments were applied in the lab. Rhizosphere samples were collected through washing roots and centrifugation with sterile PBS, with the remaining roots were collected as root samples [31]. For the indoor pot assay sampling, roots of sugarcane and maize were collected from four independent seedlings for each treatment as described above. A total of four biological replicates were collected from each N treatment in both the field trial and indoor pot culture experiment. Then, total DNA was extracted from rhizosphere soil and roots using the PowerSoil DNA Isolation Kit according to instructions in the manual (Mobio Laboratories, Carlsbad, CA, USA). V5-V7 regions of 16S rRNA were amplified using total DNA in PCR assays using primers 799F (5´-AACMGGATTAGATACCCKG-3′) and 1193R (5´-ACGTCATCCCCACCTTCC-3′) [32]. Products were further purified with the QIAquick Gel Extraction Kit (Qiagen, Germany). High-throughput sequencing was carried out at Majorbio Bio-pharm Technology Co., Ltd (Shanghai, China) using a Miseq PE300 platform (Illumina, San Diego, USA).

### Bioinformatics analysis

Raw 16S rRNA sequencing data from the Miseq PE300 platform were analyzed in the QIIME2 software platform [33]. Raw data were demuxed and trimmed using q2-demux and cutadapt [34]. The lowest quality for the base pairs was set at 25. Paired end reads were joined using vsearch [35]. A feature table was generated and classified according to the SILVA_138 gene database at a 97.0% similarity threshold [36]. R (version: 4.03) and R packages (Vegan package (version: 2.5.6)) were used for PCoA and ANOSIM analysis. The online Linear discriminant analysis effect size (LEfSe) program (http://huttenhower.sph.harvard.edu/galaxy/) was used to investigate biomarkers in different groups. The LEfSe analysis used Kruskal–Wallis rank sum test were run to test for significant differences between groups based on a threshold value of .05.

### Bacterial strain isolation and identification

Roots of sugarcane plants were collected from LN-grown sugarcane seedlings. Tissues were mashed with mortar and pestle in 10-ml sterile PBS at a laminar flow cabinet. Then PBS solution with released microorganisms were diluted 10^2^ or 10^4^ times with sterile PBS. The final solutions were plated to solid culture medium of JNFb (malate as carbon source), SNX (sucrose as carbon source), TSB (tryptic soy broth medium), and M715 (mannitol as carbon source), and placed in a 28°C incubator [37–40]. Single colonies were picked from solid culture medium and further purified by streaking to a new medium plate. Streaked isolates were further cultured in respective liquid culture medium until OD_600_ reached 1.0. Isolates were then stored at −80°C after mixed with 40.0% glycerol. To identify N-fixing bacteria, the marker gene *nifH* was selected based on its potential N-fixing capacity for PCR reactions using the primers *nifH*-F (5´-AAAGGYGGWATCGGYAARTCCACCAC-3′) and *nifH*-R (5´-TTGTTSGCSGC-RTACATSGCCATCAT-3′) [41].

Sequences of 16S rRNA fragments used for strain taxonomic identification according to a previous published method [42]. After sequencing, sequences were BLAST searched against other 16S ribosomal RNA sequences in the Bacteria and Archaea database maintained by NCBI (https://blast.ncbi.nlm.nih.gov/Blast.cgi). For phylogenetic tree construction, multiple sequence alignment was carried out with ClustW [43] and the Neighbor-Joining model [44] was used for phylogenetic tree construction (Extended Data 1). iTOL v4.0 (https://itol.embl.de/) [45] was used for tree visualization.

### Mucilage exudation under different nutrient deficiency conditions

For different nutrient deficiency treatments, nitrogen, potassium, phosphate, calcium, magnesium, and iron deficiency nutrient solutions were prepared as described [29]. Sugarcane (one-month) or maize seedlings (two-week after germination) were grown in their respective nutrient deficiency solutions using hydroponic system for two weeks. Respective seedlings of sugarcane or maize grown in normal nutrient solution acted as controls. The formation of mucilage (which were firmly attached on the tips and could not be easily removed by shaking) at the root tips was calculated and observed with a stereomicroscope (Zeiss Axio Zoom.V16) after 10 days of treatment.

### Mucilage collection and nitrogenase activity determination

For root mucilage collection, seedlings of sugarcane and maize were cultivated in their respective LN and HN hydroponic solutions for seven days. Then, mucilage formed on root tips (or nutrient solution drops on root tips) of sugarcane were collected through pipetting. Nitrogenase activity of collected mucilage was measured through acetylene reduction assay (ARA) with small modification from a previous published method [46]. To be specific, 100 μl of mucilage collected from the root tips under LN supply conditions was added to a 20-ml sterile serum vial with 5 ml Dobereiner’s N-free liquid medium, then 10.0% of the total air in the vial was replaced with C_2_H_2_. After incubation at 30°C for 12 h, the gas in the vial was analyzed with gas chromatograph (Agilent Technologies 6890 N). For control, liquid solutions (could be removed easily by shaking) were collected from root tips of sugarcane grown under HN conditions and heat-pretreated mucilage (100°C for 10 min) were included as control and negative-control treatments, respectively.

### GFP and GUS labeling

For the generation of green fluorescent protein- (GFP) and GUS-labeled microbes, competence cells of microbes were prepared according to a previous published method. Vectors expressing GFP, *pBBR1MCS2-pAmp-EGFP*, or GUS (glucuronidase), *pBBR1MCS2-pAmp-GUS* [47] were transformed into competence cells of microbes through electrotransformation performed according to a previous published method [26]. Positive colonies were identified using kanamycin antibiotic-resistance testing and fluorescence or GUS staining of micro colonies.

### Tissue colonization analysis

For tissue colonization analysis, GUS-labeled microbes were cultured in respective culture medium supplemented with 50 μg/ml kanamycin antibiotic until OD_600_ reached 1.5. GUS-labeled bacteria were collected by centrifugation and re-suspended with nutrient solutions to an adjusted OD_600_ = 1.0 before use. Then, 50 ml of prepared bacterial solution was added to 2 L HN or LN nutrient solution and co-cultured with sugarcane seedlings as described above. Colonization patterns of microbes on root tissues were determined through GUS staining after five days of co-culturing with GUS-labeled microbes [29]. Colonization patterns were recorded with a stereomicroscope (Zeiss Axio Zoom.V16).

### Mucilage metabolite analysis

Mucilage formed on root tips of sugarcane seedlings growing in LN conditions was collected through pipetting as described above. Collected mucilage was filtered through 0.22-μm filters to remove microorganisms prior to freeze drying at 4°C (Labconco FreeZone Plus 2.5 L, America), and finally conducting a nontargeted metabolic analysis at Novogene Institute (Beijing, China). Raw data were generated by a Xevo G2-XS QTOF (Waters, UK) mass spectrometer, and analyzed with Progenesis QI (version 2.2) (Waters, UK) and the R package metaX [48, 49]. Metabolites were detected under both positive and negative ion patterns.

To measure BA concentrations in sugarcane and maize mucilage under HN and LN conditions, high-performance liquid chromatography (HPLC, Waters) was employed with a C_18_ column using methanol and ammonium acetate (5:95) acting as the mobile phase at 1 ml/min flow rate, and the BA signal detect at 230 nm [50].

### Microbial chemotaxis assay

To visualize microbial chemotaxis in the proximity of metabolites or mucilage, metabolites were first selected based on abundance in mucilage. Each metabolite was dissolved in low-boiling point agarose (0.4%) with the final concentration of metabolite set to .01% after mixing gently. Then, 20 μl of agarose supplemented with metabolite was added to the center of a microscope slide where GFP-labeled microbes suspended in the sterile water (OD_600_ = 0.3) were added around the agarose [51]. After covering with cover glass, the slide was observed in the GFP channel for chemotaxis analysis at indicated time points. Pictures were taken at the starting point (0 min) and 30 min after slide preparation. The accumulation of GFP signal besides the agar indicated chemotaxis of microbes to corresponding metabolite. The density of GFP fluorescence was proportional to bacteria abundance, and density of GFP fluorescence were determined with imageJ software [52].

### Microbial inoculation and plant growth promotion assay

To test bacterial strains for plant growth promotion effects, candidate stains were first cultured in TSB culture medium to an OD_600_ value of 1.5. Then, 200 ml of bacterial culture solution was centrifuged at 8000 rpm for 20 min. Bacterial pellets were then washed twice and suspended in hydroponic culture solution to an OD_600_ of 0.2. Fifty ml of prepared inoculant solution was injected into each culture pot (7 × 7 × 14 cm) filled with sterile vermiculite, and planted with sugarcane, seedlings at the four-leave stage or seeds of maize. For indoor testing, sugarcane and maize seedlings were co-cultured with selected strains in pots for 30 days. In field tests, seedlings were co-cultured with bacterial strains indoors for 15 days before transplanting seedlings combined with vermiculite into the field in 5-m long plots of 0.8-m interspaced rows of seedlings planted every 0.5 m. A total of three plots were planted for each treatment. Plants were grown in the field for three months.

### Spray culture assay

To investigate possible functions for mucilage during colonization of sugarcane roots by N-fixing *Paraburkholderia*, sugarcane plants were first grown in HN or LN nutrition solution for 10 days prior to being transferred to the spray culture pots where 5 μl of GFP-labeled SUR17 (OD_600_ = 1) was inoculated to root tips. After two days of growth in spray culture pots, roots previously inoculated with GFP-labeled SUR17 were sampled and subjected to microscopic observation in the GFP channel. Intensity of GFP signaling on roots was proportional to the abundance of SUR17.

### Genome sequence and assembly

Genomic DNA of SUR17 was extracted using the EasyPure® Bacteria Genomic DNA Kit (TRANs GEN, Beijing) according to instructions in the manual. Extracted DNA was subjected to whole genome sequencing and assembly by Biomarker Co. Ltd (Shanghai City). The Oxford Nanopore Technologies (ONT) system was used for SUR17 genome DNA sequencing according to ONT standard protocols, including Qubit and 0.3% agarose gels used for DNA concentration and integrity detection, and the BluePipin automated nucleic acid recovery system used for DNA recovery. SQK-LSK109 kit was used for library construction. For assembly of the SUR17 genome, sequence data was assembled with Canu v1.5 and then corrected with Racon v3.4.3, before completing sequence circulation with Circlator v1.5.5. Finally, high-throughput sequencing data were used for sequence correction using Pilon v1.22 [53–55]. After the assembly of the whole genome, Prodigal v2.6.3 [56] was used for gene prediction, and annotation with the NR database [57] and Kyoto Encyclopedia of Genes and Genomes (KEGG) database [58]. Finally, the average nucleotide identity (ANI) value was calculated using the online service of JSpecies (https://jspecies.ribohost.com/jspeciesws/) according to the instructions [59].

### Benzoic acid tolerance assay

To test the benzoic acid (BA) tolerance of *Paraburkholderia* SUR-17, *E. coil* DH5a was selected as a reference bacterium. Both *Paraburkholderia* SUR-17 and *E. coil* DH5a were cultured in TY liquid culture medium, respectively. Then, each microbe was suspended using sterile water and adjust to OD_600_ = 0.5 before mixing 500 μl of *Paraburkholderia* SUR-17 and *E. coil* DH5α with 100 ml of new TY liquid medium supplemented with 0, 5, 20, 20, or 30 mM of BA and incubating at 28°C, shaking at 200 rpm. Three replicates were prepared for each BA concentration treatment. The OD_600_ values of bacterial suspensions were measured at 3, 6, 9, 17, 21, 25, 30, 40, 50, and 60 time (hours) subsequent to initiating treatment for the growth curve generation.

### Nitrogenase activity measurements from plant tissue

Roots of sugarcane and maize were harvested and cut separately into pieces with sterile scissors (0.5 cm). Then, 0.10 g of root tissue was placed in a 30 ml culture bottle with 5 ml JNFB culture medium before sealing and co-culture at 30°C for 48 h. After that 1 ml air was drawn from each sealed bottle and 1 ml pure acetylene gas was reinfected back into the bottle and incubated at 30°C for another 24 time (hours). After reaction, 1 ml 50.0% trichloroacetic acid (TCA) was injected into the vial to termination reaction. Then, the air in the vial was detected using a gas chromatograph (Agilent Technologies 6890 N). Finally, the nitrogenase activity were calculated [nmol (C_2_H_4_)·g^−1^(FW)·h^−1^] [60] = [C × (V1−V2)]/(M × FW × H). C is the concentration of ethylene on the standard curve. V1 is the volume of vial. And V2 is the volume of sample. M represents the molecular mass of ethylene. FW, the fresh weight of sample. H represents the reaction time (hour) with ethyne.

### *NifH* gene quantification

To quantify *nifH* gene copy numbers, total DNA was extracted from soil treated with BA in the microcosm assay using the same methods as outlined for extracting total soil DNA. The primer pair *nifH*-F: AAAGGYGGWATCGGYAARTCCACCAC and *nifH*-R: TTGTTSGCSGCRTACATSGCCATCAT was used for gene amplification. A standard copy number line was generated using *nifH* gene copy number dilutions of 10^−7^, 10^−6^, 10^−5^, 10^−4^, 10^−3^, and 10^−2^. The qRT-PCR runs were performed with SYBR Green in 15 μl reaction system: 7.5 μl 2 × SYBR Green Mix, 0.7 μl forward and reverse primers (10 μM), 1 μl DNA, 5.8 μl ddH_2_O. The PCR reaction program was 95°C 5 min，and 40 cycle of 95°C for 20 s, 53°C for 20 s，72°C for 40 s，then 95°C for 10 s，60°C for 60 s，95°C for 30 s, and 95°C for 15 s to complete the melting curve. Copy numbers of the *nifH* gene were calculated with Ct values and the standard curve [61].

### Microcosm assay

To test whether BA could be used for manipulating soil microbiomes, soils were collected from the sugarcane experimental field station of Fujian Agriculture and Forestry University. After sieving, 5 g soil was loaded into a sterile 5-ml sterile centrifuge tube. For the BA treatment, 200 μl of 500 μM BA was added to 5 g soil in tubes every 5 days and incubated at 20°C. For the control treatment, an equal amount of sterile water was added. Soils were sampled at 15 days and 30 days after BA addition. The composition of bacteria and the change of bacteria in tested soils were determined through 16 s rRNA genes sequencing as previously described.

### Associative ^15^N_2_ fixing assay

For the investigation the associative NF capacity, the 2 L (500 ml per week) of ^15^N_2_ gas (Re’er Info Tech Co., Ltd, Shanghai, China) with 99.0% of purity was inject to the hermetic growth chamber (100 × 80 × 80 cm), the condition for plant grown were the same with previously described in this study. The sugarcane or maize plants were inoculated (+SUR17) or noninoculated (−SUR17) with associative N fixating bacteria SUR17 under LN supply conditions. The inoculation procedure was as previous describe. For the validation the function of BA in promoting NF in maize, maize plants were grown using soils pretreated or nonpretreated with BA as previous described. Plants were grown in the hermetic growth chamber supplemented with ^15^N_2_ gas. Both sugarcane and maize plant were grown in the ^15^N_2_ gas chamber for 30 days. Then, the plants were harvested and dried in an oven for two days at 60°C and ground into powder using a hammer mill. Samples were analyzed for total N content and ^15^N enrichment using a Thermo Finnigan Delta plus Advantage Mass Spectrometer coupled with an elemental analyzer by Shanghai Research Institute of Chemical Industry Co. Ltd. (Shang Hai, China) detail for ^15^N enrichment analysis according to a previous published method [62].

### Measurement of plant biomass and N content

Plants were harvested and the biomass was determined as dry weight according to a previous published method [39]. To measure N contents, dried plants were grinded to powder prior to digesting about 0.1–0.2 g and measuring total N content using a continuous flow analyzer (SAN++). Procedures and computational analysis according to a previous published method [63].

### Statistical analysis

Means and SE values were calculated using SPSS software version 19 [64]. Duncan’s multiple comparison was used to calculate significance among samples. The two-tailed student’s *t* test was used to calculate the significance between two samples. Permutation testing was used to analyze differences at the community level. The analysis of 16 s rRNA sequence data was performed in the QIIME2 platform in Linux [33]. All statistical analyses were performed using SPSS software version 19, GraphPad Prism version 7.0 (GraphPad Software Inc., San Diego, CA, USA; https://www.graphpad.com) and R (version: 4.0).

## Results

### LN drives specific enrichment of *Burkholderia* and *Paraburkholderia* in sugarcane

As sugarcane was discovered with high ANF capacity [19], and because this knowledge has already led to successful applications in Brazilian sugarcane plantations [20], we therefore used sugarcane to investigate how N supply influences ANF.

**Figure 1:**
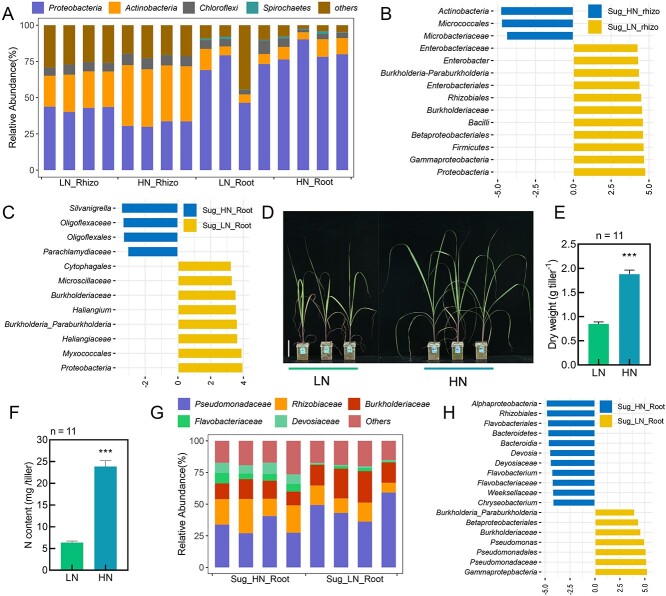
LN drives enrichment of *Paraburkholderia*_*Burkholderia* in sugarcane. A, Composition analysis of dominate bacteria at the phylum taxonomic level in the rhizosphere and root compartments of sugarcane grown under HN or LN field conditions. **B-C**, Linear discriminant analysis (LDA) score of bacterial biomarkers in the rhizospheres (**B**) and roots (**C**) of sugarcane grown in HN and LN treatments under field conditions **d**, Pictures of sugarcane seedlings grown under HN and LN conditions in an indoor pot culture assay. Bar = 10 cm. **E-F**, Biomass (**E**), and nitrogen content (**F**) of sugarcane in **D**. Error bars indicate mean + SE. *n* = 6. **G**, RA of bacterial community members at the family level in the roots of maize grown under HN and LN conditions in an indoor pot culture assay. **H**, Biomarkers in the roots of maize under HN and LN growth conditions according to LDA score. Graphed taxa are those with LDA score > 3.5 (**B**, **C**, and **H**) and Kruskal–Wallis rank sum test *P* < .05. HN_Root: roots grown in high nitrogen; LN_Root: roots grown in low nitrogen; HN_rhizo: rhizospheres in high nitrogen treatment; LN_rhizo: rhizospheres in low nitrogen treatment. Asterisk(s) (in **E**-**F**) indicate significant differences with respect to the control group at the 5% (*), 1% (**), and 0.1% (***) levels in the Student’s *t* test.

Microbiota investigation was carried out in root-associated compartments (roots and rhizoplane) of sugarcane (elongation stage) grown in HN and LN field conditions. Here, *Proteobacteria* (41.2%) and *Actinobacteria* (22.6%) were the dominant bacteria in the rhizosphere, and *Proteobacteria* (68.5%) was the dominate bacteria in the roots (Fig. 1A). In addition, N treatment affected bacterial communities in both rhizosphere and root compartments of sugarcane (Fig. 1A; Supplementary Fig. S1). Further analysis revealed that some microbes were significantly influenced by N status in both of the tested compartments, with relative abundance (RA) of *Burkholderia_Paraburkholderia* significantly enriched in the rhizosphere and roots of sugarcane plants under LN conditions (Fig. 1B and C; Supplementary Fig. S2A and B). Further experimentation with sugarcane in indoor pot cultures returned sugarcane plants with growth significantly inhibited in the LN treatment, as evident in slower plant growth, lower biomass accumulation, and LN content (Fig. 1D-F). Consistent with previous observation in the field trial, the *Burkholderiaceae* and *Burkholderia_Paraburkholderia* taxon groups were significantly enriched in the roots of pot-cultured sugarcane (Fig. 1G and H; Supplementary Fig. S2C). Taken together, these results suggest that bacterial communities associating with roots of sugarcane may be significantly altered according to N status, with *Burkholderia_Paraburkholderia* being specifically enriched in the rhizospheres and roots of sugarcane under LN stress.

**Figure 2:**
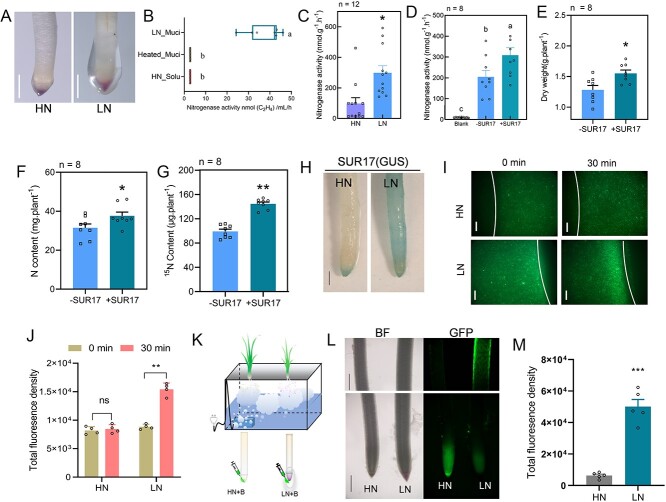
Root tip mucilage recruits nitrogen-fixing *Paraburkholderia* to sugarcane. **A**, Pictures of mucilage formation on the root tips of sugarcane seedlings grown under HN or LN hydroponic conditions. Bars = 0.2 cm. **B**, Nitrogenase activities in mucilage formed on root tips of sugarcane under nitrogen deficiency (LN_Muci) conditions in comparison with HN root tip solution (HN_Solu) and heat-treated mucilage (Heated_Muci) treatments. *n* = 6. **C**, Nitrogenase activities in the root tissues of sugarcane under HN and LN conditions. *n* = 10. **D**, Nitrogenase activity in the sugarcane roots inoculated with –SUR17 or +SUR17 treatments, along with blank control. Data are mean + SE. *n* = 9. **E-G**, The dry weight (**E**), total N content (**F**), and ^15^N content (**G**) of sugarcane plants grown with (+SUR17) or without (−SUR17) SUR17 inoculation in the grown chamber supplemented with ^15^N_2_ gas. **H**, GUS staining of sugarcane roots colonized by SUR17 under N sufficiency (HN) and deficiency (LN) conditions. Bars = 2 mm. **I**, Chemotaxis analysis of GFP-labeled SUR17 to the mucilage formed under low nitrogen conditions (LN) using an agarose-in-plug bridge assay. Solution drops collected from tips of high nitrogen-treated roots (HN) were collected to serve as control. Pictures were taken at 0 and 30 min after the addition of bacterial solution to the micro-slide. **J**, GFP fluorescence density in B were quantified with image J software. **K**, Diagram of the spray culture assay and the inoculation of GFP-labeled SUR17 to root tips. **L**, Colonization of GFP-labeled SUR17 in roots of sugarcane previously grown under HN or LN conditions using the spray culture assay in **K**, with micrographs taken in bright field and GFP channels two days after inoculation. Density of GFP was proportional to the abundance of GFP-labeled RBCS17. Bars = 0.2 cm. **M**, Density of GFP signal in the mature zone of roots in **L** quantified with image J software. Different letters (in **B** and **D**) indicate significant differences among different treatments in Duncan’s multiple comparisons test. Asterisk(s) (in **C**, **E, F, G,** and **M**) indicate significant differences with respect to the control group at the 5% (*), 1% (**), and 0.1% (***) levels in the Student’s *t* test.

Changes in the bacterial communities inhabiting root-associated compartments of sugarcane in response to N status was observed in concert with substantial mucilage formation on root tips of LN but not HN-treated sugarcane (Fig. 2A). This mucilage formation was further confirmed to be a specific response of sugarcane to N deficiency by failing to observe it in phosphorus (P), potassium (K), magnesium (Mg), Calcium (Ca), and Iron (Fe) deficiency treatments (Supplementary Fig. S3). As previous studies suggested that the formation of mucilage on adventitious roots can be considered a representative trait of BNF in monocot crops [7, 27, 65–68]. We thusly measured nitrogenase activity in the mucilage around sugarcane root tips. Nitrogenase activity as detected in acetylene reduction assays was conspicuous in the mucilage of sugarcane but not in the blank control or heat-treated mucilage (Fig. 2B). This result suggested that mucilage formation driven by LN in sugarcane might be associated with NF.

Because N-fixing bacteria are essential for ANF in sugarcane plants [19], and we observed specific enrichment of *Burkholderia_Paraburkholderia* in root-associated compartments of sugarcane under LN conditions (Fig. 1), we determined the composition of bacteria in sugarcane root mucilage. Among all bacteria identified in sugarcane root mucilage, *Burkholdriaceae* exhibited the highest RA (Supplementary Fig. S4), and nitrogenase activity was significantly higher in LN- than in HN-treated roots (Fig. 2C). We then isolated microbes from roots of sugarcane plants grown under LN condition using both N-free and N-containing culture media (Supplementary Fig. S5). In total, 779 isolates were obtained and taxonomic assignment through 16 s rRNA sequencing showed isolates were mostly *Burkholderia*, *Pseudomonas*, *Pantoea*, *Enterobacter*, *Bacillus*, and *Sphingomonas* taxa (Supplementary Fig. S6A; Supplementary Table 1). Further inspection revealed a total of 104 potential N-fixing isolates, with 17 isolates assigned to a *Paraburkholderia*/*Burkholderia* genus (Supplementary Fig. S6B; Extended Data Table 1).

With the significant enrichment of *Paraburkholderia*/*Burkholderia* genera in the roots of LN-treated sugarcane, and the high frequency of *Paraburkholderia*/*Burkholderia* taxa carrying the *nifH* gene in sugarcane root isolates, we speculated that sugarcane might recruit and enrich N-fixing *Paraburkholderia*/*Burkholderia* to deal with N deficiency. Therefore, candidate *Paraburkholderia*/*Burkholderia* for pot-based plant-microbe interaction assays were selected on the basis of carrying the *nifH* gene. A variety of growth promoting effects were observed among sugarcane exposed to the different isolates of *Paraburkholderia*/*Burkholderia* (Supplementary Fig. S7). The isolates SUR17, SUR21, SUR114, and SUR133 were selected for further field testing based on their superior growth promoting effects on sugarcane in pots. In field results, SUR17 was associated with the highest sugarcane growth promotion and N acquisition impacts (Supplementary Fig. S8).

We then labeled SUR17 with GUS or GFP tags to assess colonization (Supplementary Fig. S9). Extensive observation of GUS labeling indicated that SUR17 is able to colonize compartments across sugarcane roots (Supplementary Fig. S10). We also evaluated SUR17 application for effects on the N-fixing capacity of sugarcane. Nitrogenase activity was significantly higher in the roots of SUR17 inoculated sugarcane than in—SUR17 inoculation and negative control treatments (Fig. 2D). In addition, ^15^N^2^ enrichment assay was carried out and showed that SUR17 inoculation significantly increased biomass and total N content and also promote ^15^N integration to sugarcane plants (Fig. 2E-G). Furthermore, our results also showed that the HN treatment significantly inhibited SUR17 colonization of sugarcane roots (Fig. 2H). Based on the results above we speculated that mucilage might be involved in recruiting N-fixing *Paraburkholderia*. A chemotaxis assay with GFP-labeled SUR17 returned clear evidence of chemotaxis towards mucilage of sugarcane roots sampled from LN-treated plants and not towards roots sampled from the HN treatment (Fig. 2I and J). These results indicated that mucilage might be involved in recruiting and enhancing colonization of N-fixing *Paraburkholderia*/*Burkholderia* in the vicinity of sugarcane roots. Consistently, GFP signals emanated from entire root systems of LN-treated sugarcane, but only from tips of HN-treated roots in a spray culture assay designed to test for promotion of SUR17 colonization across host plant root systems (Fig. 2K-M). Taken together, these results suggest that N deficiency might drive sugarcane to enhance ANF by recruiting and enriching N-fixing *Paraburkholderia*/*Burkholderia* around sugarcane roots through increases in mucilage production.

### BA is a key molecule for recruiting and enriching N-fixing *Paraburkholdeia*

**Figure 3:**
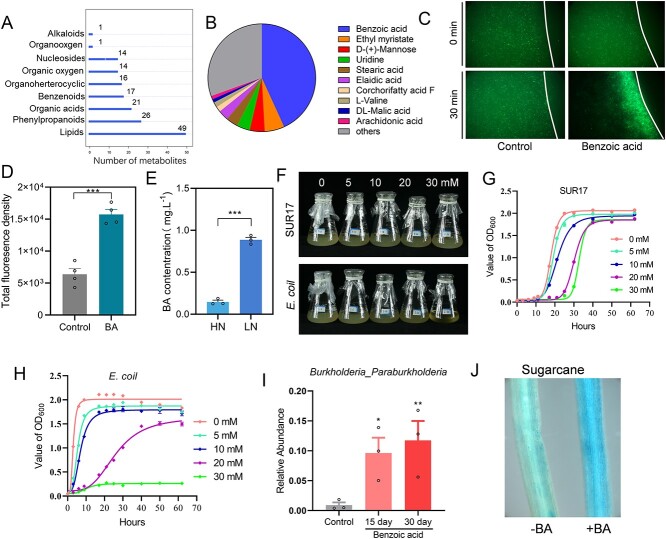
Benzoic acid is responsible for recruiting SUR-17 and inhibiting other microbes. **A**, The classification and metabolites in the mucilage formed under LN conditions were analyzed using LC–MS/MS set to measure a negative ion pattern. **B**, The relative abundance of dominate metabolites in the mucilage identified in a positive ion pattern. **C**, Chemotaxis analysis of GFP-labeled SUR17 to benzoic acid in comparison to starting time point and 30 min after initiating chemotaxis testing. White lines indicate the edge of agar. **D**, Density of GFP signaling between dashed line and solid line were quantified with image J software to measure the chemotaxis of GFP-labeled microbes towards the tested metabolites. **E**, Concentration of benzoic acid in the mucilage of sugarcane roots exposed to HN and LN treatments. Data are mean + SE. *n* = 3. **F**, Pictures of SUR17 and *E. coil* in liquid culture medium supplemented with various benzoic acid additions. (**G**, **H**). Growth curves of *Paraburkholderia* SUR17 (**G**) or *E. coil* DH5α (**H**) in **F**, with OD_600_ values of bacteria measured at the indicated time points. Data are mean + SE. *n* = 9. **I**, Relative abundance of *Burkholderia*_*Paraburkholderia* in the soil bacterial community after treatment with 500 μM benzoic acid for 0, 15, and 30 days. **J**, Colonization of GUS-labeled SUR17 on the roots of sugarcane and maize reared under –BA and +BA conditions. The blue color is proportional to the abundance of SUR17 on the roots of host plants. Asterisk(s) in (**D**, **E,** and **I**) indicate significant differences with respect to the control group at the 5% (*), 1% (**), and 0.1% (***) levels in the Student’s *t* test.

To further investigate how sugarcane mucilage recruited N-fixing *Paraburkholderia*/*Burkholderia*, we determined the composition of metabolites within mucilage using LC–MS/MS. Negative ion patterns indicated the presence of 159 metabolites in mucilage. Among identified metabolites, the most abundant was BAs, which accounted for up to 43.0% of total metabolite RA (Fig. 3A and B). In addition, 210 metabolites were also identified based on positive ion patterns (Supplementary Fig. S11). Furthermore, metabolites found in relatively high abundances were further selected for chemotaxis assays using GFP-labeled SUR17. Results revealed obvious chemotaxis of GFP-labeled SUR17 towards only a specific set of metabolites, including BA, D-mannose, phenylalanine, D-fructose, and maltose (Fig. 3C and D; Supplementary Fig. S12). In addition, results further verified that the concentration of BA in HN root tip drops was significantly lower than in LN mucilage (Fig. 3E). Taken together, based on the relative high abundance of BA and chemotaxis in recruiting SUR17, we speculated that BA might act as a potential signaling molecule in enriching N-fixing *Paraburkholderia* across sugarcane root systems experiencing LN stress.

Whole genome sequencing of SUR17 identified two contigs (Supplementary Fig. S13). Annotation of these contigs further revealed the presence of an island of genes involved in NF in the genome of SUR17 (Supplementary Fig. S14). Genome sequence and ANI is 95.6% between *Paraburkholderia* SUR17 and its closed *Paraburkholderia kururiensis* M130. [69] Through metabolic pathway analysis using the KEGG database, we identified a series of relevant pathways encoded in the genome of *Paraburkholderia* SUR17, including a benzoate degradation pathway (Supplementary Fig. S15), suggesting that SUR17 might be capable of degrading BA.

BA is typically considered an inhibitor of bacterial growth, with BA and its derivatives commonly used as preservatives in food production [70, 71]. We, therefore, speculated that BA might not only be involved in recruiting N-fixing *Paraburkholderia*, but could also inhibit the growth of other microbes. Therefore, we tested and compared tolerance of SUR17 and *E. coil* DH5α to BA. Results showed that *Paraburkholderia* SUR17 propagated normally in growth medium supplemented with up to 30 mM BA, whereas *E. coil* DH5α was significantly inhibited by exposures to 20 and 30 mM BA (Fig. 3F-H). In addition, a microcosm assay further confirmed that BA applications increased the RA of *Burkholderia*_*Paraburkholderia* in soils, whereas applying BA enhanced colonization of GUS-labeled SUR17 on the roots of sugarcane (Fig. 3I and J). Taken together, our results suggest that BA is involved in specific recruitment and enrichment of N-fixing *Paraburkholderia*, and simultaneously acting to inhibit the growth of other bacteria.

### Maize and sugarcane employ similar strategies to enrich N-fixing *Paraburkholdeia*

**Figure 4:**
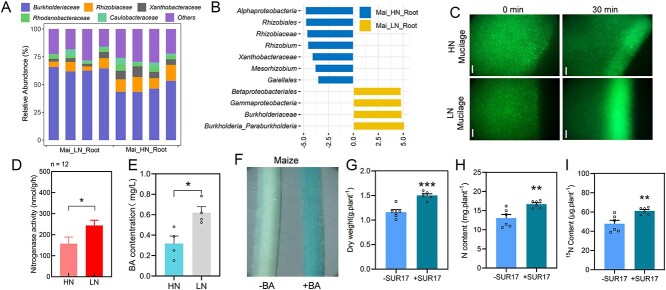
Maize employs a similar strategy to sugarcane in recruiting nitrogen-fixing bacteria. **A**, Relative abundance of bacterial community members at family level in the roots of maize reared under HN and LN conditions in an indoor pot culture assay. **B**, Biomarkers in the roots of maize under HN and LN growth conditions according to LDA score (LDA score > 3.5). **C**, Chemotaxis analysis of GFP-labeled SUR17 movement towards the mucilage of maize formed under LN and HN conditions using as observed in an agarose-in-plug bridge assay. **D**, Nitrogenase activities in the root tissues of maize under HN and LN conditions. **E**, Concentration of benzoic acid in root tip mucilage of sugarcane under HN and LN conditions. Data are mean + SE. *n* = 3. **F**, Colonization of GUS-labeled SUR17 on the roots of sugarcane and maize grown in –BA and +BA treatments. The blue color is proportional to the abundance of SUR17 on the roots of host plants. **G**-**I**, The dry weight (**G**), total N content (**H**), and ^15^N content (**I**) of sugarcane plants grown under (+SUR17) or without (–SUR17) SUR17 inoculation in the grown chamber supplemented with ^15^N_2_ gas. Data are mean + SE. *n* = 6. Asterisk(s) in (**D**, **E**, **G**, **H,** and **I**) indicate significant differences with respect to the control group at the 5% (*), 1% (**), and 0.1% (***) levels in the Student’s *t* test.

To test whether other C_4_ crops act similarly to sugarcane in recruiting N-fixing bacteria, we ran further experiments with maize, the most widely cultivated monocot crop. Results showed N treatment also significantly altered bacterial communities in the roots of maize (Supplementary Fig. S16) (ANOSIM, *R* = 1.00, *P* = .02), for which *Burkholderiaceae* and *Burkholderia_Paraburkholderia* were significantly increased by LN treatment in comparisons with HN treatment (Fig. 4A and B). GUS-labeled SUR17 could also colonized roots of maize (Supplementary Fig. S17). Also, GFP-labeled SUR17 exhibited chemotaxis towards LN-derived mucilage, but not HN derived mucilage in maize (Fig. 4C). We also found more nitrogenase activity and higher BA concentrations in LN roots and LN mucilage of maize than in HN roots and HN mucilage (Fig. 4D and E). Consistently, BA treatment obviously enhanced the colonization of GUS-labeled SUR17 to the roots of maize (Fig. 4F). Inoculation of SUR17 significantly increased nitrogenase activity and promote the maize growth and N acquisition LN conditions (Supplementary Fig. S18). Finally, ^15^N_2_ isotope enrichment assay further confirmed ANF confers the integration of ^15^N to the maize plants (Fig. 4G-I). Taken together, our results suggest that maize and sugarcane employ similar strategies in recruiting N-fixing bacteria under LN conditions.

### Application of BA to enhance NF

**Figure 5:**
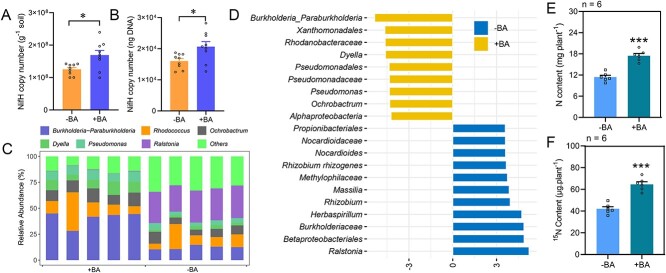
BA application increases *nifH* gene abundance in soils and enhances ANF in maize. **A-B**, Copy numbers of the *nifH* gene in the –BA (0 Μ) and +BA (500 μM) benzoic-acid-treated soils per g soil (**A**) or per ng total soil DNA (**B**). Data are mean + SE. *n* = 3. **C**, Bacterial composition analysis at the genus taxonomic level from the roots of maize grown in soil under –BA or +BA conditions. **D**, Bacterial biomarkers from roots of maize under –BA and +BA condition, with LDA score > 3.5 and Kruskal–Wallis rank sum test *P* < .05. **E** and **I**, The total N content (**E**) and (**I**) of maize plants grown under (+SUR17) or without (-SUR17) SUR17 inoculation in the grown chamber supplemented with ^15^N_2_ gas. Data are mean + SE. *n* = 6. Asterisk(s) in **A**, **B**, **E** and **F** indicate significant differences with respect to the control group at the 5% (*), 1% (**) and 0.1% (***) levels in the Student’s *t* test.

To further test whether the application of BA could increase the abundance of N-fixing bacteria, we employed a microcosm assay. Results showed that addition of BA significantly increases the abundance of *nifH* gene copy number in tested soils (Fig. 5A and B). In addition, the RA of *Paraburkholderia*/*Burkholderia* was higher in BA pretreated soils than in the—BA soil treatment (Fig. 5C and D). Overall, we found that maize grew more vigorously, with higher biomass accumulation, and higher N content in +BA than in –BA treatments (Supplementary Fig. S17). In addition, BA application combined with ^15^N_2_ isotope enrichment assay showed BA application promoted N acquisition and enhanced ANF in maize (Fig. 5E and F). Taken together, these results suggest that BA application may potentially increase abundance of N-fixing bacteria and copy number of the *nifH* gene in soils, which combine to enhance ANF in and around host plants.

## Discussion

N-fixing bacteria associated with monocot crops have drawn considerable interest over the last century due to the potential for this association to be utilized in efforts to reduce reliance on N fertilizers and simultaneously enhance environmental protection [72–75]. However, the molecular mechanisms underlying associative NF in monocot crop systems have remained largely mysterious. In this study, we determined how monocot crops specifically recruit and enrich N-fixing bacteria from soil using mucilage and specific metabolites that are responsive soil N supplies.

Here, the C_4_ crops sugarcane and maize were studied in a wide range of experiments designed to elucidate how they manifest high-potential ANF capacities. Bacterial communities in root-associated compartments were influenced by soil N status in both sugarcane and maize reared in field or pot culture assays (Figs 1A, G, 4A), which is consistent with recent reports of N effects on sugarcane and maize root microbiomes [46, 76–78]. Furthermore, the RA of *Paraburkholderia*/*Burkholderia* was significantly higher in LN than in HN treatments for both sugarcane and maize (Figs 1B, C, H and4B; Supplementary Fig. S2). Selection and application of *Paraburkholderia*/*Burkholderia* strains carrying *nifH* led to increases in N acquisition in both sugarcane and maize (Fig. 2E-G; Fig. 4G-I; Supplementary Figs S7–S8, 18).

Previous studies suggest that *Paraburkholderia*/*Burkholderia* may serve as a specific component of core sugarcane microbiota [46, 79, 80]. Previous studies have also shown that *Paraburkholderia*/*Burkholderia* is a beneficial class of endophytes for sugarcane, maize, wheat, and rice [81–88]. In this study, this taxon was identified through16s rRNA high-throughput sequencing, which pegged it not only as highly abundant in root-associated compartments of sugarcane and maize, but also as significantly up-regulated in response to low soil N conditions (Figs 1B, C, 4B; Supplementary Fig. S2). This is consistent with recent results in which *Paraburkholderia*/*Burkholderia* was identified as an important taxon in associations with maize [78]. It is worth noting that no response to N treatment was observed for this N-fixing taxon in a similar study [77], which might be due to differences in the soil types or sugarcane genotypes investigated among the set of available experimental results [89].

High N supplies significantly inhibited nitrogenase activity in our observations (Fig. 2C and d), which is consistent with previous reports of HN inhibition of NF [90, 91]. Further investigation showed that LN treatment induced the formation of mucilage on sugarcane root tips (Fig. 2A; Supplementary Fig. S3), though the mechanisms backing the regulation of mucilage formation by N availability remains unknown [7, 92]. Consistent with the stimulation of N-fixing bacteria and NF in mucilage on aerial roots of wheat, barely, sorghum, and maize identified in previous reports [7, 27, 93–95], in our experiments, we observed conspicuous nitrogenase activity and high abundances of *Burkholderiaceae* in sugarcane mucilage (Fig. 2B, Supplementary Fig. S4). This suggests similarities between sugarcane and other monocot crops. Moreover, we also demonstrated that either LN treatment or mucilage from LN treatments could enhance recruitment and colonization of N-fixing *Paraburkholderia* SUR17 on the roots of sugarcane and maize (Figs 2E-J, E).

Previous studies have suggested that mucilage provides a high-nutrient and low-oxygen environment for ANF that is similar to the conditions under which SNF manifests in rhizobial colonized nodules on legume roots [7, 27, 96, 97]. Other studies suggest that mucilage forms on root tips to protect root caps through immobilization of toxic ions such as aluminum [98, 99]. In our results, mucilage on root tips induced by LN was also involved in root microbiome establishment. In addition, the results outlined here for mucilage activities on plant roots are similar to those described for mucilage in animal guts, where mucilage on intestinal surfaces regulates associations with gut microbiota [100], as well as to those reported for mucilage roles in the symbiosis between *Gunnera* and *Nostoc* species, where mucilage also stimulates symbiotic partners whereas simultaneously excluding other bacteria [101, 102]. Recently, a study on *Heterotis rotundifolia* reveal that the aerial root-mucilage and the microbiota living in it confer the associative NF [103]. In conclusion, here we demonstrated that monocot crops appear to employ strategies for recruiting ANF microbes that are similar to those utilized in other plant species, and which are consistent with roles filled by mucilage components in both plants and animals.

The molecular and regulatory pathways underlying specific enrichment of N-fixing *Paraburkholdeia* by sugarcane remains unknown. Previous investigations of the composition of maize mucilage identified a variety of carbohydrates that can act as energy sources for dizotrophic microbiota living in the mucilage [7, 27, 104, 105]. Here, we instead detected high concentrations of BA in sugarcane and maize mucilage (Fig. 3A and B), with the concentration of BA also induced by LN treatment in both sugarcane and maize (Figs 3E and  4D). These results, suggest that BA levels might be commonly regulated by N status across in monocot crops, which needs further investigation [106]. Further results demonstrated that BA in the mucilage was involved in recruiting N-fixing *Paraburkholderia* SUR17 (Fig. 3C and D), but not as a primary energy source, because SUR17 was not able to utilize BA as its sole carbon source (Data not shown). Similarly, recent report in rice suggests specific flavone metabolites confer the recruitment of diazotrophic bacteria [107]. These results indicate that BA, which was identified as an allelochemical in a previous study, might act as a signaling molecule in recruiting N-fixing *Paraburkholderia*. [50]

Previous work has also suggested that BA can be an important secondary metabolite released by plants to deal with soil-borne pathogens as either a signaling molecule [108] or an antimicrobial agent [109]. In our research, genome sequencing and annotation suggested that *Paraburkholderia* SUR17 may harbor the potential to degrade benzoate (Supplementary Fig. S14), whereas experimentation demonstrated that *Paraburkholderia* SUR17 tolerates higher concentrations of BA in growth media than *E. coil.* (Fig. 3F-H). This is consistent with previous reports of high BA degradation capacities in *Paraburkholderia*/*Burkholderia* taxa [110, 111]. This strategy of incorporating normally toxic compounds as primary signaling agents is similar to those observed with some legumes in which selection of beneficial microbes is mediated through channels that simultaneously inhibit other microbes [112, 113]. In our results, BA changed the soil microbiome through increases in the RA of *Paraburkholderia*/*Burkholderia* and copy numbers of the *nifH* gene (Figs 3I, 5A and B), which enhanced colonization by N-fixing bacteria and NF in host plants (Figs 3J, 4F, 5C, 5D), and ultimately enhances N acquisition and promotes plant growth (Supplementary Fig. S19; Fig. 5E and F). Determining whether BA might perform similar functions in C_3_ monocot crops, such as rice and wheat, requires further investigation [114].

**Figure 6:**
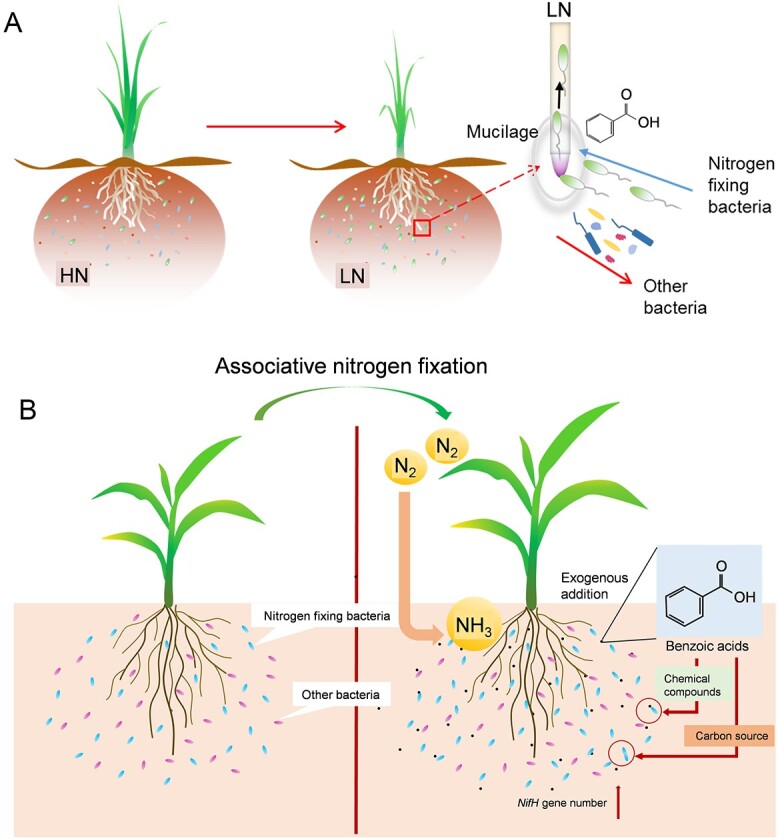
Models for the *Paraburkholderia* enrichment and a strategy for enhancing ANF in monocot crops. **A**, Model of interactions between nitrogen-fixing *Paraburkholderia* and cereal crops. When graminaceous crops are challenged with low nitrogen conditions, nitrogen deficiency induces the formation of mucilage on root tips, with high amounts of benzoic acid accumulating in the mucilage. The accumulation of mucilage attracts nitrogen-fixing *Paraburkholder*/*Burkholderia* to the mucilage, whereas simultaneously inhibiting the growth of other bacteria, and, thusly, increases the relative abundance of nitrogen-fixing *Paraburkholder*/*Burkholderia* in mucilage and promotes the colonization of nitrogen-fixing *Paraburkholder*/*Burkholderia* to the roots of graminaceous crops, which increases the adaptability of graminaceous crops to the low nitrogen conditions. HN: high nitrogen; LN: low nitrogen. **B**, Model for enhancing ANF through application of BA in monocot crops. BA application changes soil bacterial communities and increases *nifH* gene abundance, which further enhances ANF in cereal crops.

In conclusion, we propose a potential model for interactions between monocot crops and associative N-fixing *Paraburkholderia*/*Burkholderia* bacteria in which BA application can enhance ANF in monocot crops. Mucilage formation on sugarcane root tips in response to LN stress, and BAs is involved in the recruitment of N-fixing *Paraburkholderia*/*Burkholderia* from soils, simultaneous inhibition of other bacteria. This combination of effects leads to the specific selection of N-fixing *Paraburkholderia*/*Burkholderia* by mucilage and enhanced colonization of these beneficial partners on the roots of plants experiencing LN stress (Fig. 6A). In addition, BA could increase the RA of N-fixing *Paraburkholderia*/*Burkholderia*, as well as, increases in the abundance of *nifH* gene copies, which enhances associative NF and improves N acquisition in monocot crops exposed to LN growing conditions (Fig. 6B). On the whole, our investigations identified a regulatory mechanism employed by C_4_ crops to manipulate associated microbiomes in response to N deficiency (Fig. 6A). Elucidation of the physiology and biochemistry backing this response points to new avenues involving specific signaling molecules to explore for solutions to harnessing beneficial microbes for increases in ANF in C_4_ crops (Fig. 6B) [115].

## Supplementary Material

Supplementary_figures_and_legends_wrae210

Sup_Data_1_wrae210

Sup_Tab_1_wrae210

Sup_Tab_2_wrae210

## Data Availability

Raw data supporting the conclusions of this article are deposited with NCBI: BioProject ID(PRJNA906464). The raw data for the bacteria genome sequencing and genome assembly is deposited with NCBI: BioProject ID(PRJNA944133).

## Code availability

All code from this study is available upon request.
