# Correction to: Benzoic acid facilitates ANF in monocot crops by recruiting nitrogen-fixing *Paraburkholderia*

**DOI:** 10.1093/ismejo/wrae238

**Published:** 2024-12-04

This is a correction to: Ran Liu, Ruirui Li, Yanjun Li, Mingjia Li, Wenjing Ma, Lei Zheng, Cunhu Wang, Kefei Zhang, Ya Tong, Guoqiang Huang, Xinxin Li, Xin-Guang Zhu, Chuihuai You, Yongjia Zhong, Hong Liao, Benzoic acid facilitates ANF in monocot crops by recruiting nitrogen fixing *Paraburkholderia*, *The ISME Journal*, 2024, wrae210, https://doi.org/10.1093/ismejo/wrae210

When the author's accepted manuscript version of this paper was first published, a production error caused the wrong manuscript to be uploaded. The correct version of the accepted manuscript was loaded shortly afterwards. The publisher apologizes to the authors and readers for any inconvenience caused.

