# Supplementary material for: Benzoic acid facilitates ANF in monocot crops by recruiting nitrogen-fixing Paraburkholderia
Source: ISME J. 2024 Oct 22;18(1):wrae210. doi: 10.1093/ismejo/wrae210 (PMC11632831; doi:10.1093/ismejo/wrae210)
Supplement: Supplementary_figures_and_legends_wrae210 [file supplementary_figures_and_legends_wrae210.docx]

**Supplemental Figure legends**

**
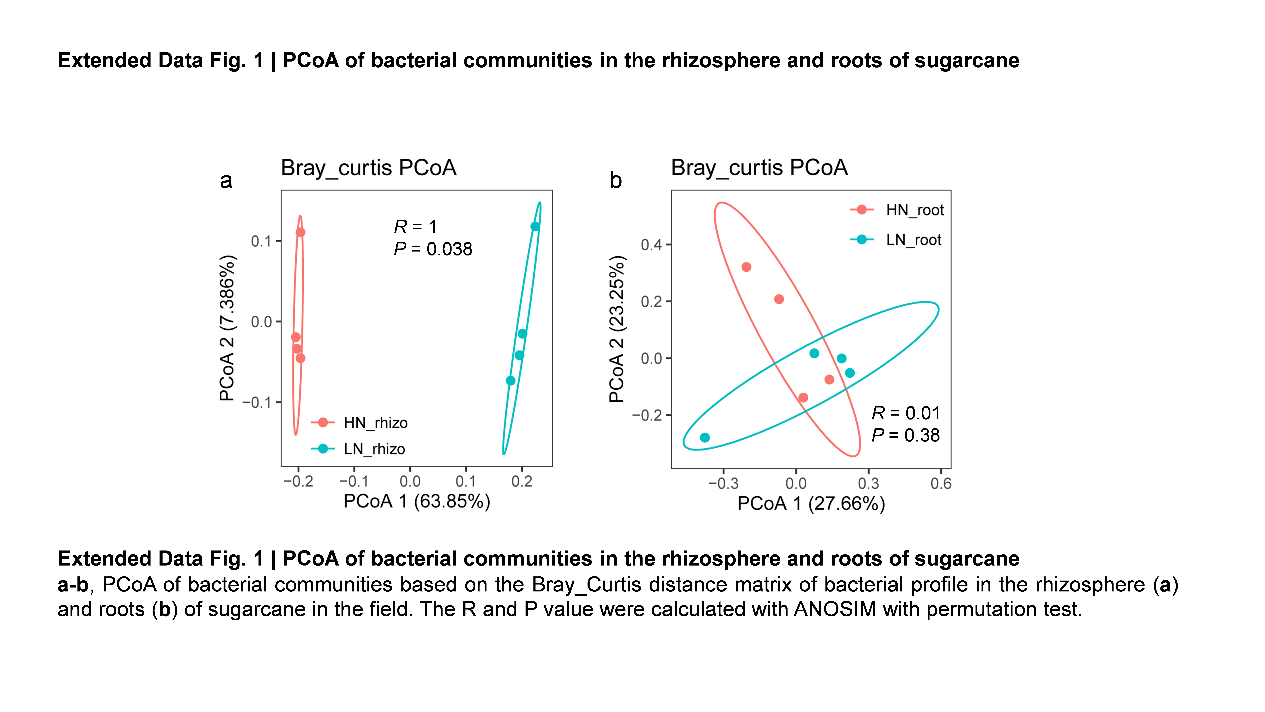
**

**Supplementary Fig. S1 PCoA of bacterial communities in the rhizosphere and roots of sugarcane**

**a** and **b**, PCoA of bacterial communities based on the Bray_Curtis distance matrix of bacterial profiles from the rhizosphere (**a**) and roots (**b**) of sugarcane grown in the field. The R and P value were calculated in ANOSIM with permutation tests.

**
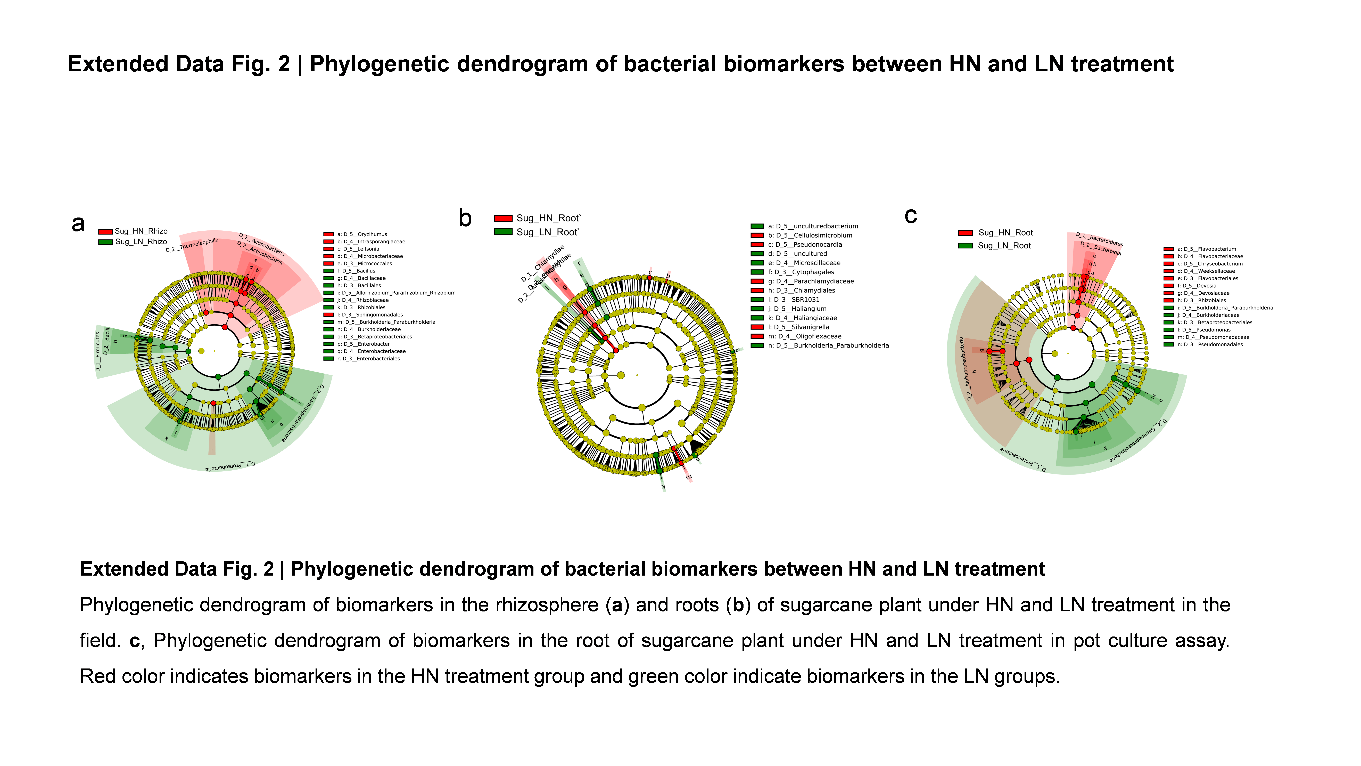
**

**Supplementary Fig. S2 Phylogenetic dendrogram of bacterial biomarkers identified in HN and LN treatments**

Phylogenetic dendrogram of biomarkers in the rhizosphere (**a**) and roots (**b**) of sugarcane plants grown in HN and LN treatments in the field. **c**, Phylogenetic dendrogram of biomarkers in the roots of sugarcane plants grown in HN and LN treatments in a pot culture assay. Red color indicates biomarkers in the HN treatment group and green color indicates biomarkers in the LN group.


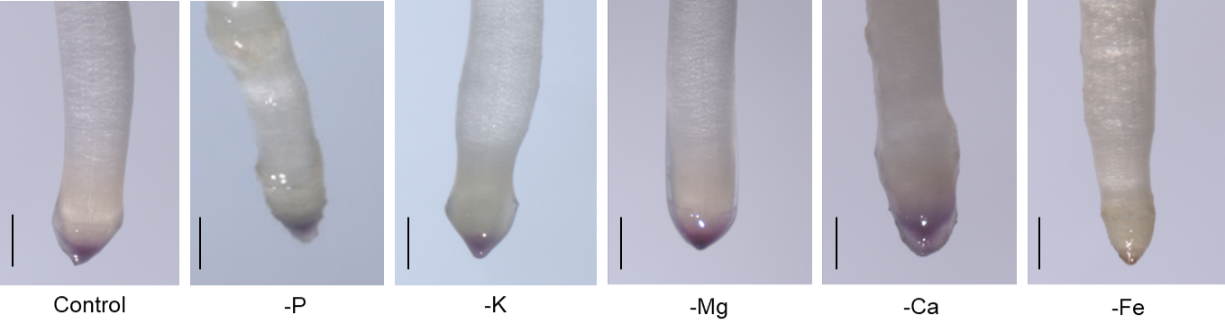


**Supplementary Fig. S3 Influence of nutrient deficiency on the formation of mucilage on the root tips of sugarcane**

Pictures of mucilage formation on the root tips of sugarcane and maize seedlings grown under different nutrient deficiency conditions. Full nutrient solution treatment act as control. Bar = 0.2 cm. P: phosphorus; K: potassium; Mg: magnesium; Ca: calcium; Fe: iron.

**Supplementary Fig. S4 Composition of bacteria in the mucilage of sugarcane**

Relative abundance of bacterial community members at the family taxonomic level for five samples of mucilage collected from root tips of sugarcane grown under LN conditions. Composition of bacterial communities were determined through 16S rRNA sequencing.


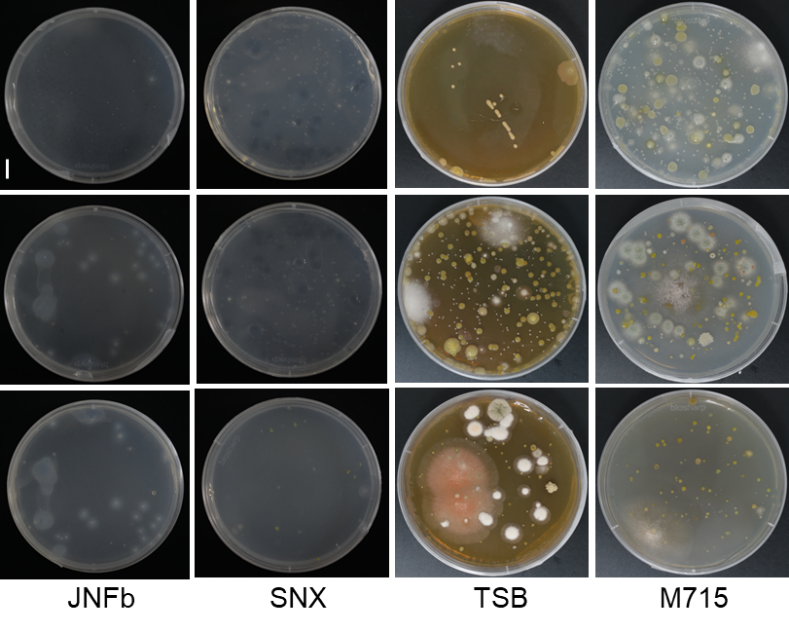


**Supplementary Fig. S5 Bacterial growth on culture medium with and without nitrogen included**

Pictures of bacteria growth on the nitrogen free (JNFb, SNX) and nitrogen containing culture medium (TSB, M715) five days after plating and incubating at 28 °C. Bar = 1 cm.


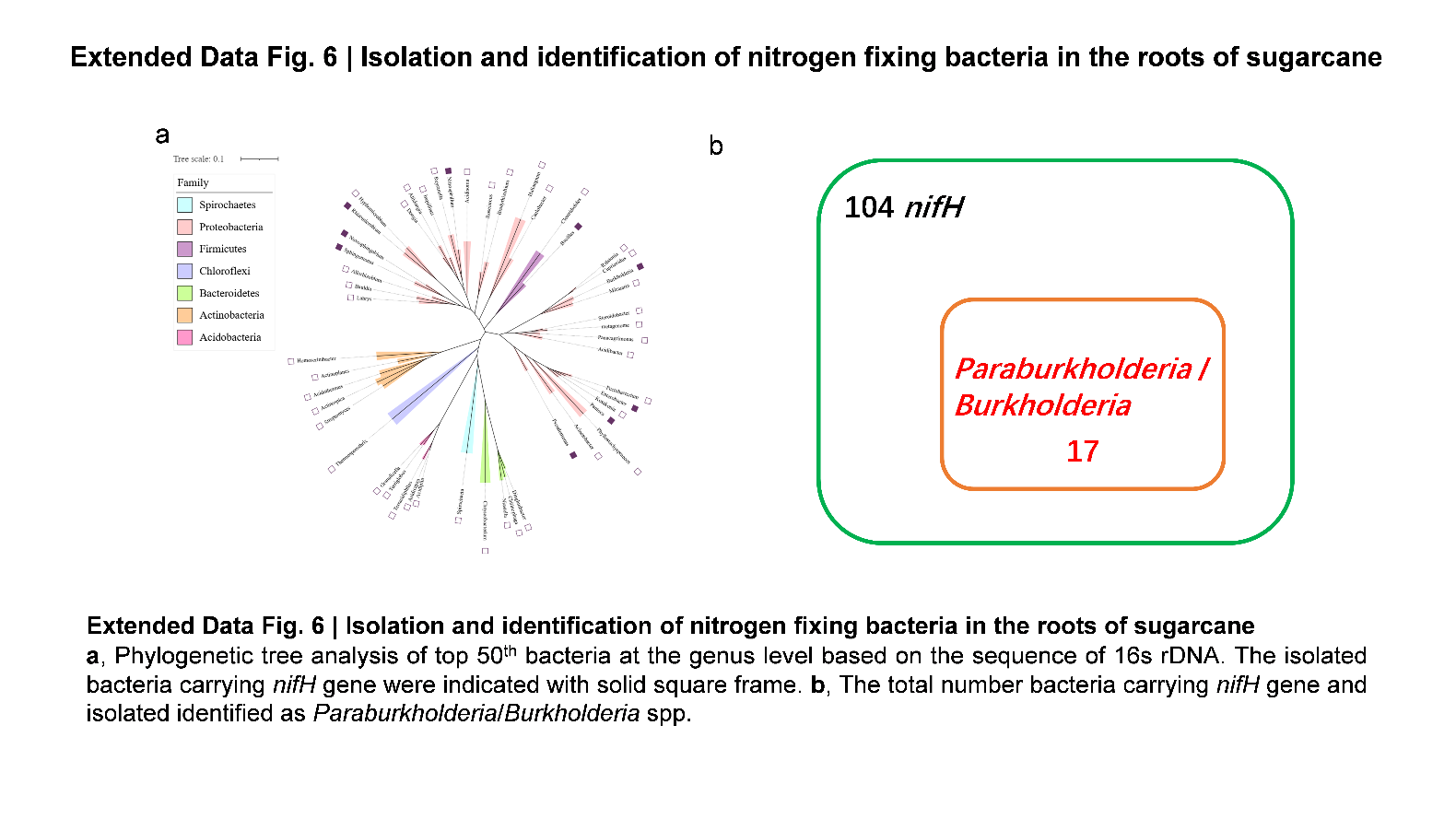


**Supplementary Fig. S6 Isolation and identification of nitrogen fixing bacteria in the roots of sugarcane**

**a**, Phylogenetic tree analysis of the top 50 bacteria at the genus level based on the sequence of 16s rRNA. Isolated bacteria carrying the *nifH* gene are indicated with a solid square frame. **b**, The total number isolated bacteria carrying the *nifH* gene and identified as *Paraburkholderia*/*Burkholderia* spp.


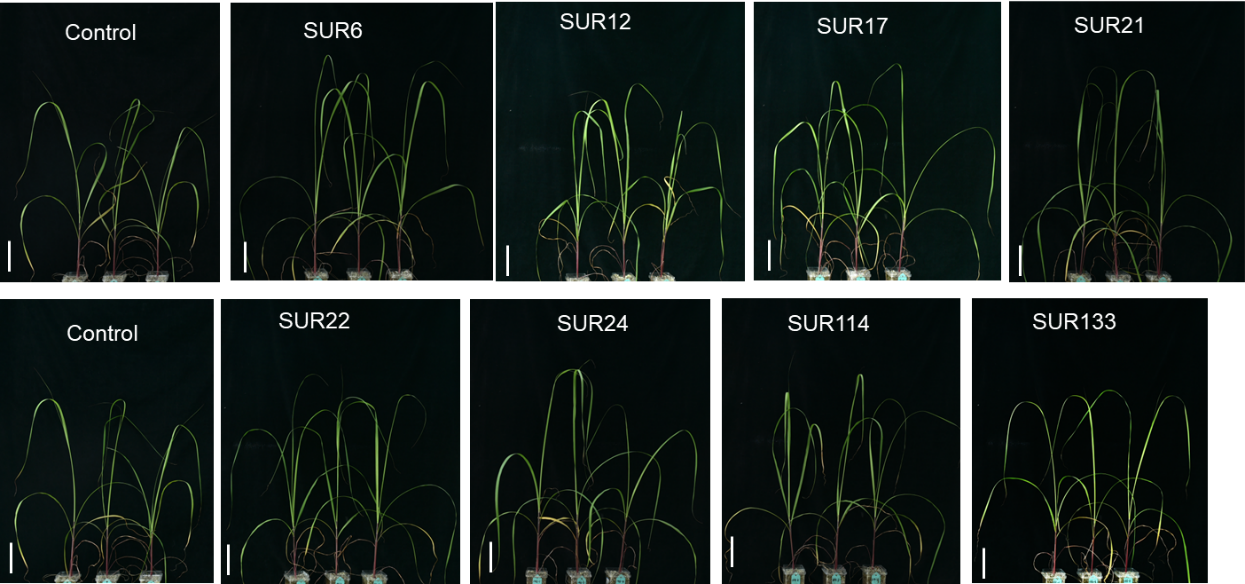


**Supplementary Fig. S7 Application of potential PGPR to the growth of sugarcane seedlings**

Comparison of sugarcane seedling growth performance in co-cultures with potential beneficial bacteria reared for 50 days in a growth chamber. Bars = 8 cm.


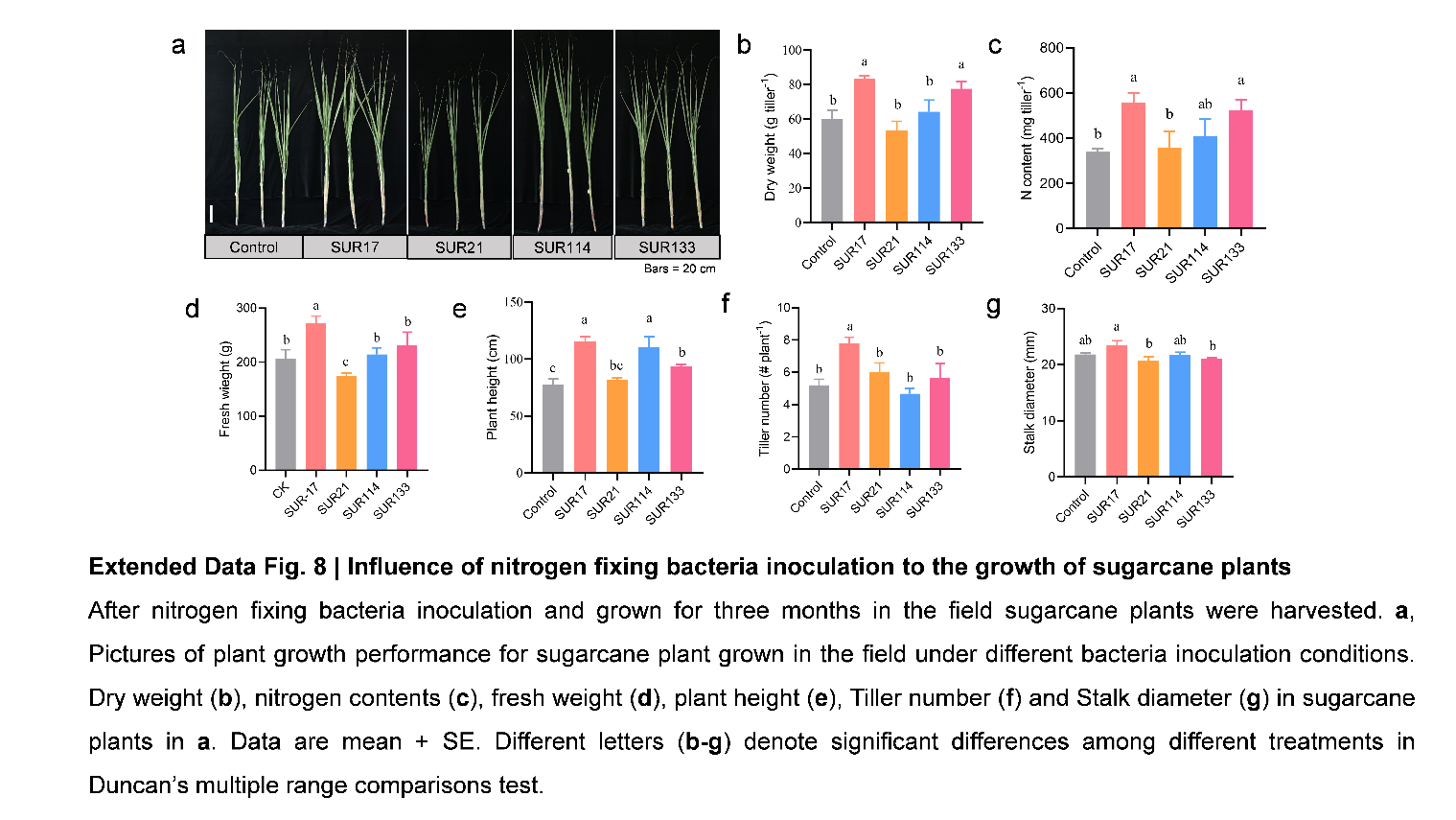


**Supplementary Fig. S8 Influence of nitrogen fixing bacteria inoculation on growth of sugarcane plants**

After inoculation of nitrogen fixing bacteria and three months of subsequent growth, sugarcane plants were harvested from field plots (non-inoculation used as control). **a**, Pictures of plant growth performance for sugarcane plants grown in the field under different bacteria inoculation conditions. Dry weight (**b**), nitrogen contents (**c**), fresh weight (**d**), plant height (**e**), Tiller number (**f**) and stalk diameter (**g**) of sugarcane plants in **a**. Data are mean + SE. Different letters (**b-g**) denote significant differences among different treatments in Duncan’s multiple range comparisons test.


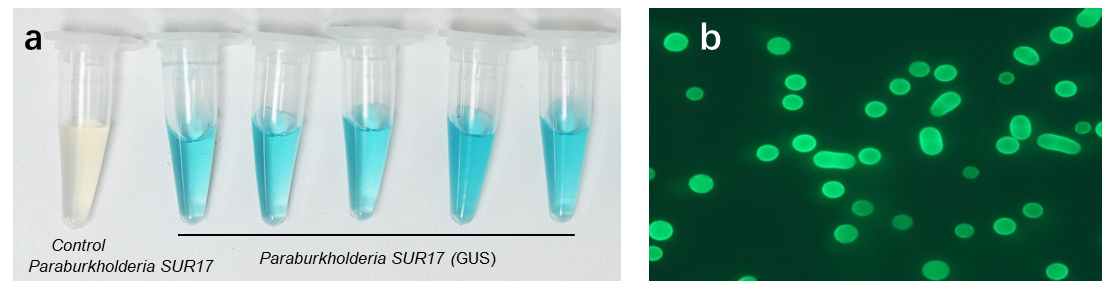


**Supplementary Fig. S9 Labeling SUR17 with GUS and GFP**

**a**, Picture of GUS staining for SUR17 labeled with the GUS marker, and unlabeled SUR17 acting as a blank control. The blue color indicates microbial cells were successfully labeled with the GUS marker gene. **b**, Picture of GFP labeled SUR17 on antibiotic amended medium plates imaged in fluorescence microscopy.


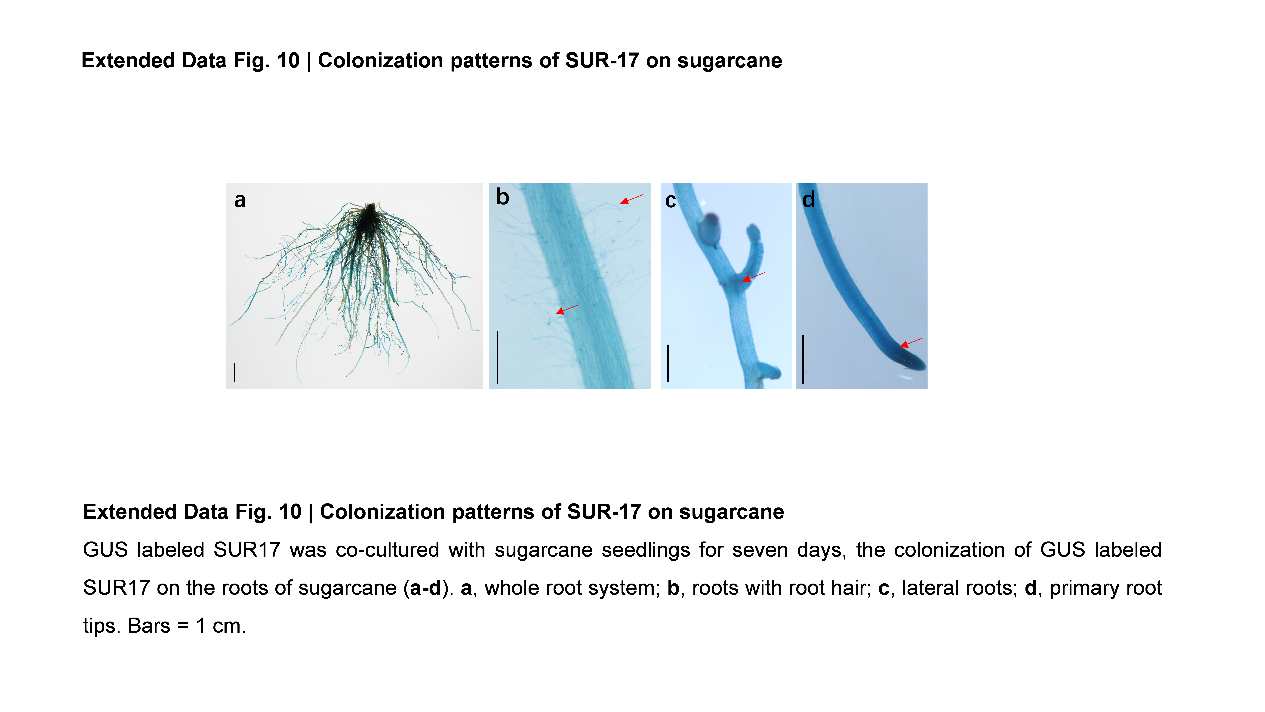


**Supplementary Fig. S10 Colonization patterns of SUR-17 on sugarcane**

GUS labeled SUR17 was co-cultured with sugarcane seedlings for seven days, the colonization of GUS labeled SUR17 on roots of sugarcane (**a-d**). **a**, whole root system; **b**, roots with root hair; **c**, lateral roots; **d**, primary root tips. Bars = 1 cm.


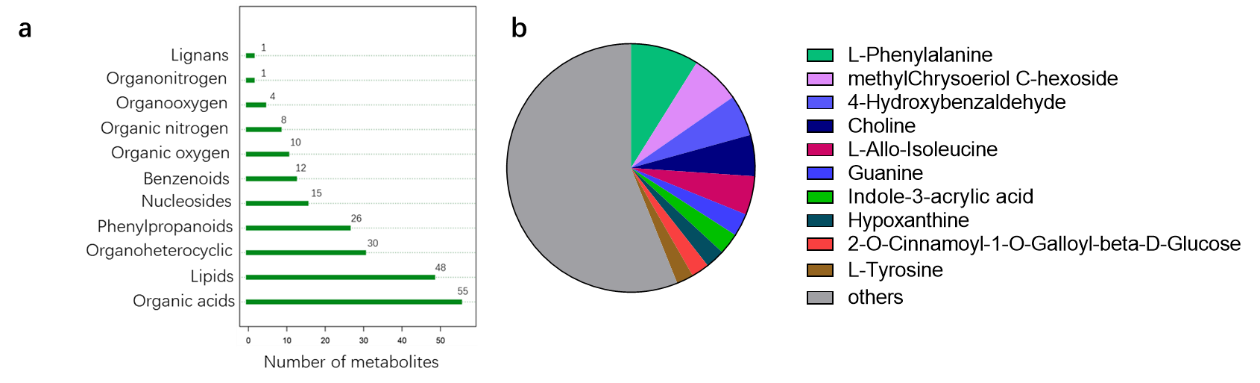


**Supplementary Fig. S11 Metabolites identified with positive ion patterns**

**a**, The classification and number of metabolites in the mucilage formed under LN condition were analyzed with LC-MS/MS using a positive ion pattern. **b**, The relative abundance of dominate metabolites in the mucilage using a positive ion pattern.


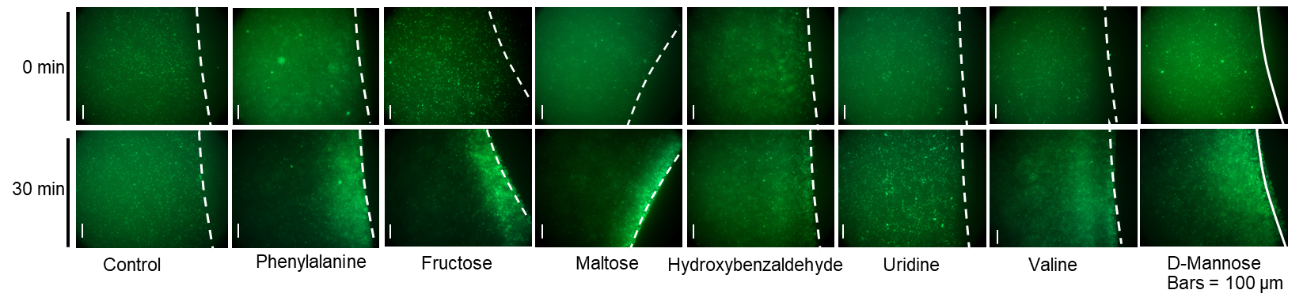


**Supplementary Fig. S12 Chemotaxis responses of SUR-17 to different metabolites**

Chemotaxis of GFP labeled SUR-17 towards different metabolites as identified through LC-MS/MS protocols as described in the methods. Pictures were taken at the start point (0 min) and 30 min after inoculation. For control, equal amount of ddH_2_O was used in the chemotaxis assay. The GFP signal enriched in the agar edge indicates that the chemotaxis of SUR-17 towards the metabolite in the agar.


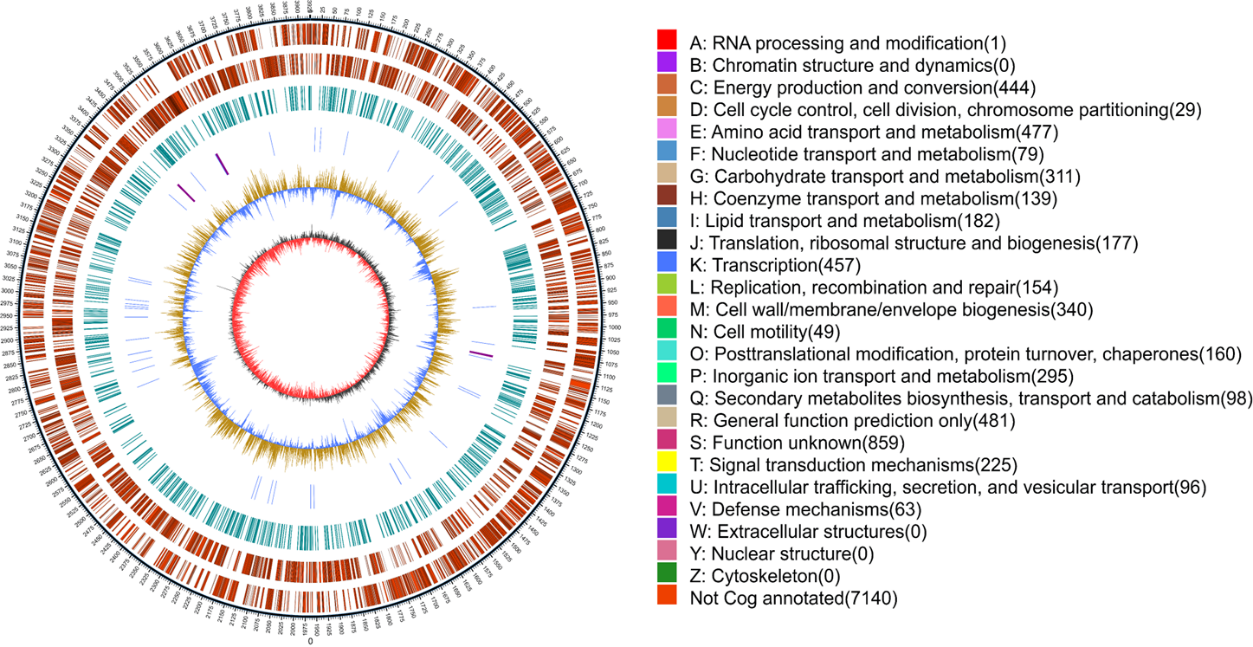


**Supplementary Fig. S13 Whole genome and genome annotation of SUR17**

Genome circus plot of *Paraburkholderia* SUR-17 using whole genome sequences and genome annotation information.


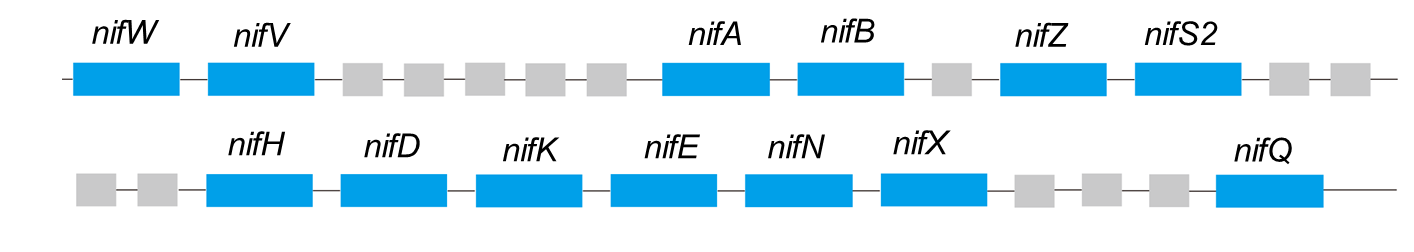


**Supplementary Fig. S14 Diagram of nitrogen fixing island on the genome of SUR17**

The nitrogen fixing island on the genome is shown and nitrogen fixation functional genes are indicated in blue and labelled.


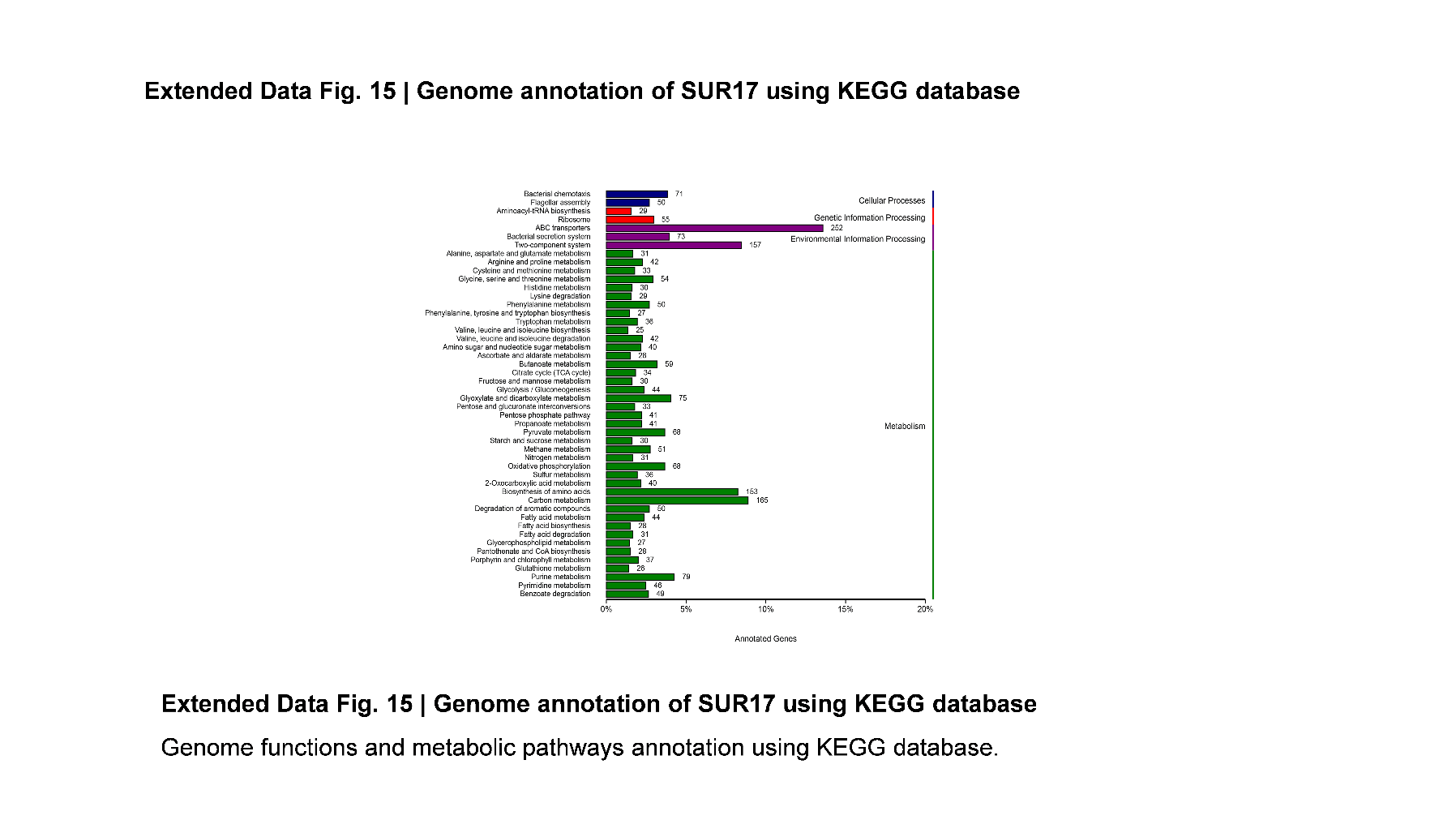


**Supplementary Fig. S15 Genome annotation of SUR17 using KEGG database**

Genome functions and metabolic pathways as annotated using the KEGG database.


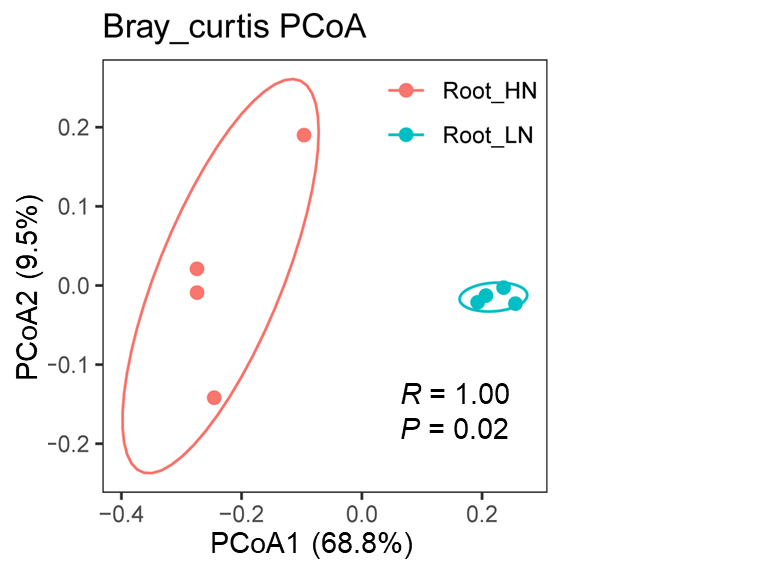


**Supplementary Fig. S16 PCoA of bacterial communities in the roots of sugarcane**

PCoA of bacterial communities based on the Bray-Curtis distance matrix of bacterial profiles in the rhizosphere of maize grown in an indoor pot culture assay. The *R* and *P* values were calculated in ANOSIM with permutation testing.


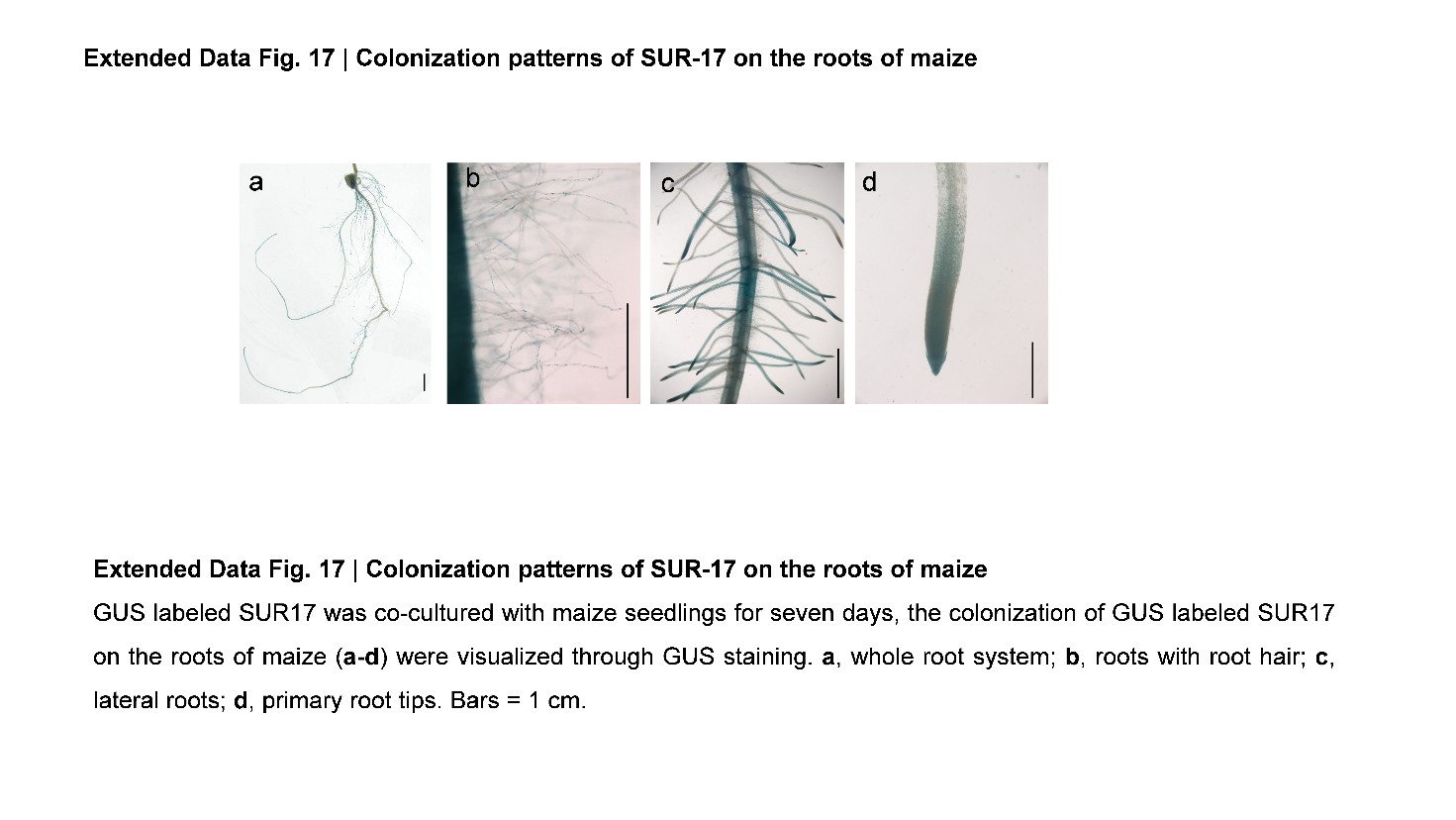


**Supplementary Fig. S17 Colonization patterns of SUR17 on maize**

GUS labeled SUR17 was co-cultured with maize seedlings for seven days, the colonization of GUS labeled SUR17 on the roots of maize (**a-d**) was visualized through GUS staining. **a**, whole root system; **b**, roots with root hair; **c**, lateral roots; **d**, primary root tips. Bars = 1 cm.


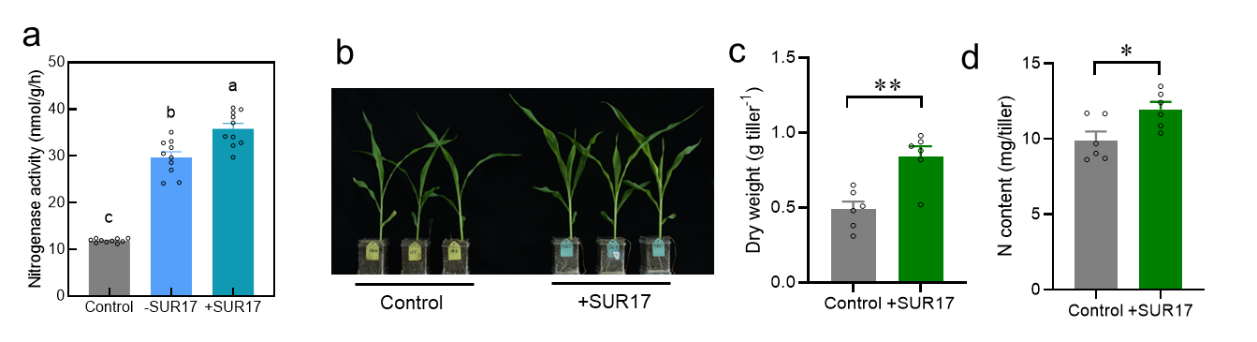


**Supplementary Fig. S18 Inoculation of SUR17 on maize plant growth and N acquisition**

**a**, Nitrogenase activity in the maize roots inoculated with –SUR17 or +SUR17 treatments, along with heat-treatment controls. Data are mean +SE. n = 10. **b**, Growth performance of maize grown under SUR17 inoculation or control (non-inoculation) solutions. (**c**, **d**). Dry weight (**c**) and total nitrogen content (**d**) of maize in **b**. Error bars represents + SE. n = 6. Different letters (in **a**) indicate significant differences among different treatments in Duncan’s multiple comparisons test. Asterisk(s) in (**c** and **d**) indicate significant differences with respect to the control group at the 5% (*), 1% (**) and 0.1% (***) levels in the Student’s *t* test.


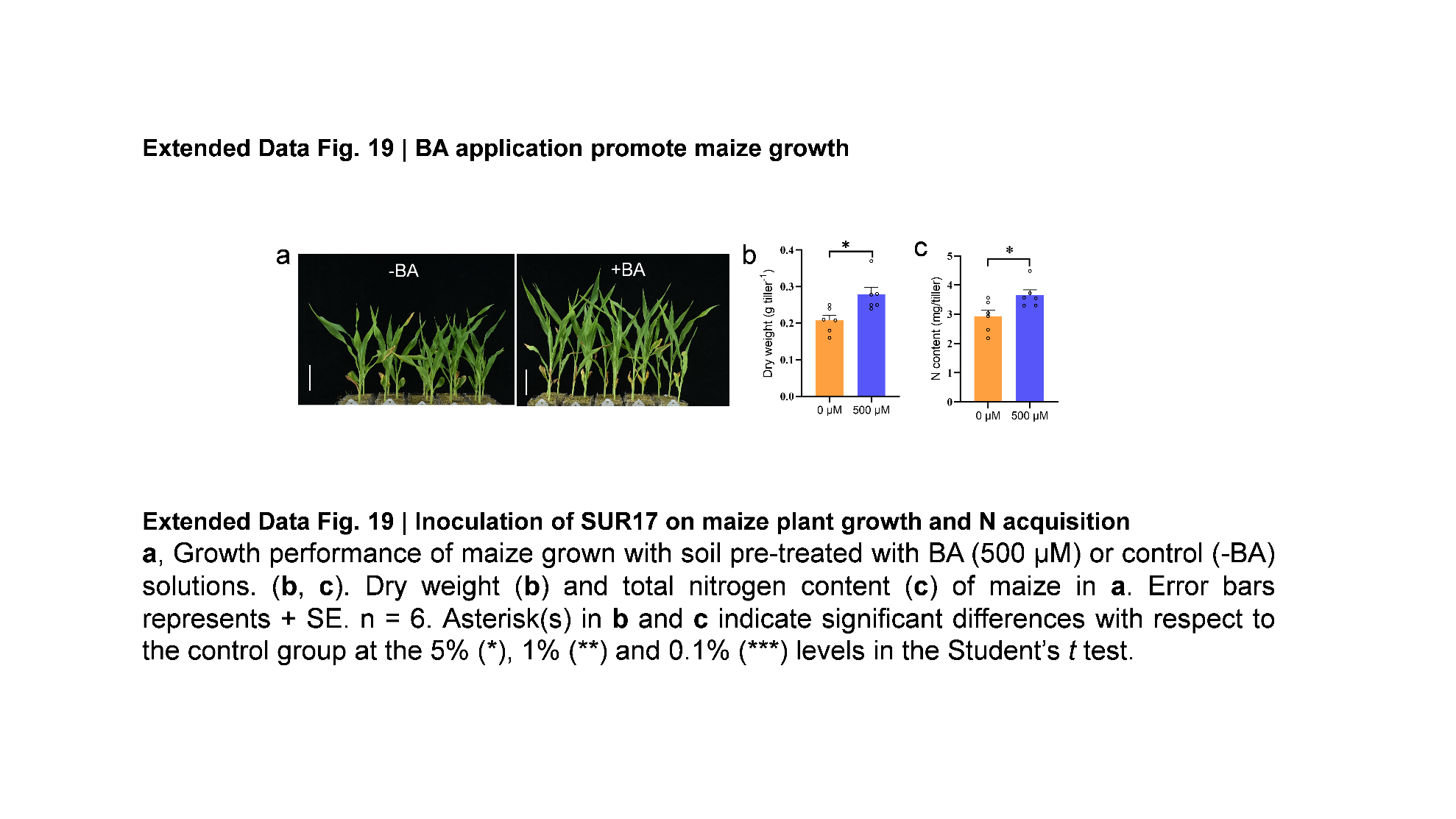


**Supplementary Fig. S19 Inoculation of SUR17 on maize plant growth and N acquisition**

**a**, Growth performance of maize grown with soil pre-treated with BA (500 μM) or control (0 μM) solutions. (**b**, **c**). Dry weight (**b**) and total nitrogen content (**c**) of maize in **a**. Error bars represents + SE. n = 6. Asterisk(s) in **b** and **c** indicate significant differences with respect to the control group at the 5% (*), 1% (**) and 0.1% (***) levels in the Student’s *t* test.
