# Supplementary material for: Benzoic acid facilitates ANF in monocot crops by recruiting nitrogen-fixing Paraburkholderia
Source: ISME J. 2024 Oct 22;18(1):wrae210. doi: 10.1093/ismejo/wrae210 (PMC11632831; doi:10.1093/ismejo/wrae210)
Supplement: Sup_Tab_2_wrae210 [file sup_tab_2_wrae210.docx]

Extended data 1. Supplemental Table 1. Basic soil characters of experimental field

| pH Value | Organic matter (g/kg) | Alkali-hydrolyzable  nitrogen (mg/kg) | Available phosphorus  （mg/kg） | Available potassium (mg/kg) |
| --- | --- | --- | --- | --- |
| 5.42±0.09 | 24.02±1.28 | 100.68±1.28 | 115.95±7.01 | 107.59±7.48 |

Supplemental Table 2. Microbes identified with nifH gene.

| Strain | Species | nifH | Nitrogenase activity | Ca-phosphate solubilization | Phytin solubilization | IAA production |
| --- | --- | --- | --- | --- | --- | --- |
| SUR-6 | *Paraburkholderia bannensis* strain BE22 | + |  | + | + |  |
| SUR-10 | *Paraburkholderia kururiensis* strain IC-76A | + |  |  |  |  |
| SUR-11 | *Paraburkholderia bannensis* strain BE22 | + |  | + | + |  |
| SUR-12 | *Paraburkholderia heleia* strain MaAR-230 | + | + | + | + | + |
| SUR-16 | *Paraburkholderia bannensis* strain BE22 | + |  | + | + |  |
| SUR-17 | *Paraburkholderia kururiensis* strain IC-76A | + | + | + | + | + |
| SUR-18 | *Burkholderia sp.* DRS9A | + |  | + | + |  |
| SUR-19 | *Burkholderia sp.* strain Y29 | + |  | + | + |  |
| SUR-20 | *Burkholderia heleia* SA41 | + |  |  |  |  |
| SUR-21 | *Paraburkholderia kururiensis* strain IC-76A | + |  |  | + |  |
| SUR-22 | *Paraburkholderia tropica* strain BE15 | + |  | + | + |  |
| SUR-24 | *Paraburkholderia tropica* strain BE15 | + |  | + | + |  |
| SUR-133 | *Burkholderia vietnamiensis* strain LMG 10929 | + |  | + | + |  |
| SUR-180 | *Rhizobium tropici* |  |  |  |  |  |
| SUR-181 | *Microbacterium* KE150457_s |  |  |  |  |  |
| SUR-182 | *Phytobacter ursingii* |  |  |  |  |  |
| SUR-183 | *Bacillus glycinifermentans* |  |  |  |  |  |
| SUR-184 | *Microbacterium* KE150457_s |  |  |  |  |  |
| SUR-216 | *Phytobacter ursingii* | + |  |  |  |  |
| SUR-220 | *Phytobacter ursingii* | + |  |  |  |  |
| SUR-240 | *Novosphingobium sediminicola* | + |  |  |  |  |
| SUR-270 | *Nitrospirillum* CP022112_s | + |  |  |  |  |
| SUR-283 | *Citrobacter bitternis* | + |  |  |  |  |
| SUR-284 | *Phytobacter ursingii* | + |  |  |  |  |
| SUR-286 | *Citrobacter bitternis* | + |  |  |  |  |
| SUR-288 | *Citrobacter bitternis* | + |  |  |  |  |
| SUR-291 | *Novosphingobium sediminicola* | + |  |  |  |  |
| SUR-302 | *Herbaspirillum seropedicae* | + |  |  |  |  |
| SUR-304 | *Citrobacter bitternis* | + |  |  |  |  |
| SUR-309 | *Pelomonas aquatica* | + |  |  |  |  |
| SUR-310 | *Rhizobium aquaticum* | + |  |  |  |  |
| SUR-311 | *Phytobacter ursingii* | + |  |  |  |  |
| SUR-315 | *Phytobacter ursingii* | + |  |  |  |  |
| SUR-316 | *Phytobacter ursingii* | + |  |  |  |  |
| SUR-317 | *Phytobacter ursingii* | + |  |  |  |  |
| SUR-318 | *Citrobacter bitternis* | + |  |  |  |  |

Supplemental Table 3. List of metabolites selected for the chemotaxis assay.

| **Positive material** | **Negative material** |
| --- | --- |
| **Organic acids and derivatives** | **Organic acids and derivatives** |
| L-Phenylalanine | L-Valine |
| L-Tyrosine | **Nucleosides, nucleotides, and analogues** |
| **Organoheterocyclic compounds** | Uridine |
| Guanine | **Benzenoids** |
| **Organic oxygen compounds** | Benzoic acid |
| 4-Hydroxybenzaldehyde | **Other** |
| D-(+)-Maltose | D-(+)-Mannose |
| D-(-)-Fructose |  |

>SUR-6

GGCGAGTGGCGAACGGGTGAGTAATACATCGGAACGTGTCCTGTAGTGGGGGATAGCCCGGCGAAAGCCGGATTAATACCGCATACGATCTACGGATGAAAGCGGGGGATCTTCGGACCTCGCGCTATAGGGGCGGCCGATGGCGGATTAGCTAGTTGGTGAGGTAAAGGCTCACCAAGGCGACGATCCGTAGCTGGTCTGAGAGGACGACCAGCCACACTGGGACTGAGACACGGCCCAGACTCCTACGGGAGGCAGCAGTGGGGAATTTTGGACAATGGGCGAAAGCCTGATCCAGCAATGCCGCGTGTGTGAAGAAGGCCTTCGGGTTGTAAAGCACTTTTGTCCGGAAAGAAATCCCTGGTCCTAATATGGCCGGGGGATGACGGTACCGGAAGAATAAGCACCGGCTAACTACGTGCCAGCAGCCGCGGTAATACGTAGGGTGCAAGCGTTAATCGGAATTACTGGGCGTAAAGCGTGCGCAGGCGGTGATGTAAGACCGATGTGAAATCCCCGGGCTCAACCTGGGAACTGCATTGGTGACTGCATCGCTTGAGTATGGCAGAGGGGGGTAGAATTCCACGTGTAGCAGTGA

>SUR-10 CGGAACGTATCCTGGAGTGGGGGATAGCCCGGCGAAAGCCGGATTAATACCGCATACGCTCTGAGGAGGAAAGCGGGGGACCTTCGGGCCTCGCGCTCAAGGGGCGGCCGATGGCGGATTAGGTAGTTGGTGGGGTAAAGGCCTACCAAGCCGACGATCCGTAGCTGGTCTGAGAGGACGACCAGCCACACTGGGACTGAGACACGGCCCAGACTCCTACGGGAGGCAGCAGTGGGGAATTTTGGACAATGGGGGCAACCCTGATCCAGCAATGCCGCGTGTGTGAAGAAGGCCTTCGGGTTGTAAAGCACTTTTGTCCGGAAAGAAATCATCGGGGCCAATACCCCTGGTGGATGACGGTACCGGAAGAATAAGCACCGGCTAACTACGTGCCAGCAGCCGCGGTAATACGTAGGGTGCGAGCGTTAATCGGAATTACTGGGCGTAAAGCGTGCGCAGGCGGTGCTGTAAGACCGATGTGAAATCCCCGGGCTTAACCTGGGAACTGCATTGGTGACTGCAGCGCTGGAGTATGGCAGAGGGGGGTGGAATTCCACGTGTAGCAGTGAAATGCGTAGAGATGT

>SUR-11 GTGGCGAGTGGCGAACGGGTGAGTAATACATCGGAACGTGTCCTGTAGTGGGGGATAGCCCGGCGAAAGCCGGATTAATACCGCATACGATCTACGGATGAAAGCGGGGGATCTTCGGACCTCGCGCTATAGGGGCGGCCGATGGCGGATTAGCTAGTTGGTGAGGTAAAGGCTCACCAAGGCGACGATCCGTAGCTGGTCTGAGAGGACGACCAGCCACACTGGGACTGAGACACGGCCCAGACTCCTACGGGAGGCAGCAGTGGGGAATTTTGGACAATGGGCGAAAGCCTGATCCAGCAATGCCGCGTGTGTGAAGAAGGCCTTCGGGTTGTAAAGCACTTTTGTCCGGAAAGAAATCCCTGGTCCTAATATGGCCGGGGGATGACGGTACCGGAAGAATAAGCACCGGCTAACTACATGCCAGCAGCCGCGGTAATACGTAGGGTGCAAGCGTTAATCGGAATTACTGGGCGTAAAGCGTGCGCAGGCGGTGATGTAAGACCGATGTGAAATCCCCGGGCTCAACCTGGGAACTGCAT

>SUR-12 TCGGCCTGGCGGCGAGTGGCGAACGGGTGAGTAATACATCGGAACATGTCCAGTAGTGGGGGATAGCCCGGCGAAAGCCGGATTAATACCGCATACGATCTGTGGATGAAAGCGGGGGATCTTCGGACCTCGCGCTATTGGGGTGGCCGATGGCGGATTAGCTAGATGGTGGGGTAAAGGCCTACCATGGCGACGATCCGTAGCTGGTCTGAGAGGACGACCAGCCACACTGGGACTGAGACACGGCCCAGACTCCTACGGGAGGCAGCAGTGGGGAATTTTGGACAATGGGGGCAACCCTGATCCAGCAATGCCGCGTGTGTGAAGAAGGCCTTCGGGTTGTAAAGCACTTTTGTCCGGGAAGAAATCCCCTGCCCTAATACGGCGGGGGGATGACGGTACCGGAAGAATAAGCACCGGCTAACTACGTGCCAGCAGCCGCGGTAATACGTAGGGTGCGAGCGTTAATCGGAATTACTGGGCGTAAAGCGTGCGCAGGCGGTGATGTAAGACCGATGTGAAATCCCCGGGCTTAACCTGGGAACTGCATTGGTGACTGCATCGCTGGAGTATGGCAGAGGGGGGTAGAATTCCACGTGTA

>SUR-16 GGATAGCCCGGCGAAAGCCGGATTAATACCGCATACGATCTACGGATGAAAGCGGGGGATCTTCGGACCTCGCGCTATAGGGGCGGCCGATGGCGGATTAGCTAGTTGGTGAGGTAAAGGCTCACCAAGGCGACGATCCGTAGCTGGTCTGAGAGGACGACCAGCCACACTGGGACTGAGACACGGCCCAGACTCCTACGGGAGGCAGCAGTGGGGAATTTTGGACAATGGGCGAAAGCCTGATCCAGCAATGCCGCGTGTGTGAAGAAGGCCTTCGGGTTGTAAAGCACTTTTGTCCGGAAAGAAATCCCTGGTCCTAATATGGCCGGGGGATGACGGTACCGGAAGAATAAGCACCGGCTAACTACGTGCCAGCAGCCGCGGTAATACGTAGGGTGCAAGCGTTAATCGGAATTACTGGGCGTAAAGCGTGCGCAGGCGGTGATGTAAGACCGATGTGAAATCCCCGGGCTCAACCTGGGAACTGCATTGGTGACTGCATCGCTTGAGTATGGCAGAGGG

>SUR-17 AGTGGCGAACGGGTGAGTAATACATCGGAACGTATCCTGGAGTGGGGGATAGCCCGGCGAAAGCCGGATTAATACCGCATACGCTCTGAGGAGGAAAGCGGGGGACCTTCGGGCCTCGCGCTCAAGGGGCGGCCGATGGCGGATTAGGTAGTTGGTGGGGTAAAGGCCTACCAAGCCGACGATCCGTAGCTGGTCTGAGAGGACGACCAGCCACACTGGGACTGAGACACGGCCCAGACTCCTACGGGAGGCAGCAGTGGGGAATTTTGGACAATGGGGGCAACCCTGATCCAGCAATGCCGCGTGTGTGAAGAAGGCCTTCGGGTTGTAAAGCACTTTTGTCCGGAAAGAAATCATCGGGGCCAATACCCCTGGTGGATGACGGTACCGGAAGAATAAGCACCGGCTAACTACGTGCCAGCAGCCGCGGTAATACGTAGGGTGCGAGCGTTAATCGGAATTACTGGGCGTAAAGCGTGCGCAGGCGGTGCTGTAAGACCGATGTGAAATCCCCGGGCTTAACCTGGGAACTGCATTGGTGACTGCAGCGCTGGAGTATGGCAGAGGGGGGTGGAATTCCACGTGTAGCAGTGAAATGCGTAGAGATGTGGAGG

>SUR-18 GCCCGGCGAAAGCCGGATTAATACCGCATACGATCTACGGATGAAAGCGGGGGATCTTCGGACCTCGCGCTATAGGGGCGGCCGATGGCGGATTAGCTAGTTGGTGAGGTAAAGGCTCACCAAGGCGACGATCCGTAGCTGGTCTGAGAGGACGACCAGCCACACTGGGACTGAGACACGGCCCAGACTCCTACGGGAGGCAGCAGTGGGGAATTTTGGACAATGGGCGAAAGCCTGATCCAGCAATGCCGCGTGTGTGAAGAAGGCCTTCGGGTTGTAAAGCACTTTTGTCCGGAAAGAAATCCCTGGTCCTAATATGGCCGGGGGATGACGGTACCGGAAGAATAAGCACCGGCTAACTACGTGCCAGCAGCCGCGGTAATACGTAGGGTGCAAGCGTTAATCGGAATTACTGGGCGTAAAGCGTGCGCAGGCGGTGATGTAAGACCGATGTGAAATCCCCGGGCTCAACCTGGGAACTGCATTGGTGACTGCATCGCTTGAGTATGGCAGAGGGGGGTAGAATTCCACGT

>SUR-19 TGTCCTGTAGTGGGGGATAGCCCGGCGAAAGCCGGATTAATACCGCATACGCTCTACGGAGGAAAGGGGGGGATCTTAGGACCTCCCGCTACAGGGGCGGCCGATGGCAGATTAGCTAGTTGGTGGGGTAAAGGCCTACCAAGGCGACGATCTGTAGCTGGTCTGAGAGGACGACCAGCCACACTGGGACTGAGACACGGCCCAGACTCCTACGGGAGGCAGCAGTGGGGAATTTTGGACAATGGGCGCAAGCCTGATCCAGCAATGCCGCGTGTGTGAAGAAGGCCTTCGGGTTGTAAAGCACTTTTGTCCGGAAAGAAAACCTCCGCCCTAATATGGTGGGGGGATGACGGTACCGGAAGAATAAGCACCGGCTAACTACGTGCCAGCAGCCGCGGTAATACGTAGGGTGCAAGCGTTAATCGGAATTAC

>SUR-20 GTCCAGTAGTGGGGGATAGCCCGGCGAAAGCCGGATTAATACCGCATACGATCTGTGGATGAAAGCGGGGGATCTTCGGACCTCGCGCTATTGGGGTGGCCGATGGCGGATTAGCTAGATGGTGGGGTAAAGGCCTACCATGGCGACGATCCGTAGCTGGTCTGAGAGGACGACCAGCCACACTGGGACTGAGACACGGCCCAGACTCCTACGGGAGGCAGCAGTGGGGAATTTTGGACAATGGGGGCAACCCTGATCCAGCAATGCCGCGTGTGTGAAGAAGGCCTTCGGGTTGTAAAGCACTTTTGTCCGGGAAGAAATCCCCTGCCCTAATACGGCGGGGGGATGACGGTACCGGAAGAATAAGCACCGGCTAACTACGTGCCAGCAGCCGCGGTAATACGTAGGGTGCGAGCGTTAATCGGAATTACTGGGCGTAAAGCGTGCGCAGGCGGTGATGTAAGACCGATGTGAAATCCCCGGGCTTAACCTGGGAACTGCATTGGTGACTGCATCGCTGG

>SUR-21 CATCGGAACGTATCCTGGAGTGGGGGATAGCCCGGCGAAAGCCGGATTAATACCGCATACGCTCTGAGGAGGAAAGCGGGGGACCTTCGGGCCTCGCGCTCAAGGGGCGGCCGATGGCGGATTAGGTAGTTGGTGGGGTAAAGGCCTACCAAGCCGACGATCCGTAGCTGGTCTGAGAGGACGACCAGCCACACTGGGACTGAGACACGGCCCAGACTCCTACGGGAGGCAGCAGTGGGGAATTTTGGACAATGGGGGCAACCCTGATCCAGCAATGCCGCGTGTGTGAAGAAGGCCTTCGGGTTGTAAAGCACTTTTGTCCGGAAAGAAATCATCGGGGCCAATACCCCTGGTGGATGACGGTACCGGAAGAATAAGCACCGGCTAACTACGTGCCAGCAGCCGCGGTAATACGTAGGGTGCGAGCGTTAATCGGAATTACTGGGCGTAAAGCGTGCGCAGGCGGTGCTGTAAGACCGATGTGAAATCCCCGGGCTTAACCTGGGAACTGCATTGGTGACTGCAGCGCTGGAGTATGGCAG

>SUR-22 TTGCACCTGGTGGCGAGTGGCGAACGGGTGAGTAATACATCGGAACGTGTCCTGTAGTGGGGGATAGCCCGGCGAAAGCCGGATTAATACCGCATACGATCTACGGATGAAAGCGGGGGATCTTCGGACCTCGCGCTATAGGGGCGGCCGATGGCGGATTAGCTAGTTGGTGAGGTAAAGGCTCACCAAGGCGACGATCCGTAGCTGGTCTGAGAGGACGACCAGCCACACTGGGACTGAGACACGGCCCAGACTCCTACGGGAGGCAGCAGTGGGGAATTTTGGACAATGGGCGAAAGCCTGATCCAGCAATGCCGCGTGTGTGAAGAAGGCCTTCGGGTTGTAAAGCACTTTTGTCCGGAAAGAAATCCCTGGTCCTAATATGGCCGGGGGATGACGGTACCGGAAGAATAAGCACCGGCTAACTACGTGCCAGCAGCCGCGGTAATACGTAGGGTGCAAGCGTTAATCGGAATTACTGGGCGTAAAGCGTGCGCAGGCGGTGATGTAAGACCGATGTGAAATCCCCGGGCTCAACC

>SUR-24 TGTAGTGGGGGATAGCCCGGCGAAAGCCGGATTAATACCGCATACGATCTACGGATGAAAGCGGGGGATCTTCGGACCTCGCGCTATAGGGGCGGCCGATGGCGGATTAGCTAGTTGGTGAGGTAAAGGCTCACCAAGGCGACGATCCGTAGCTGGTCTGAGAGGACGACCAGCCACACTGGGACTGAGACACGGCCCAGACTCCTACGGGAGGCAGCAGTGGGGAATTTTGGACAATGGGCGAAAGCCTGATCCAGCAATGCCGCGTGTGTGAAGAAGGCCTTCGGGTTGTAAAGCACTTTTGTCCGGAAAGAAATCCCTGGTCCTAATATGGCCGGGGGATGACGGTACCGGAAGAATAAGCACCGGCTAACTACGTGCCAGCAGCCGCGGTAATACGTAGGGTGCAAGCGTTAATCGGAATTACTGGGCGTAAAGCGTGCGCAGGCGGTGATGTAAGACCGATGTGAA

>SUR-133 ACCTGGTGGCGAGTGGCGAACGGGTGAGTAATACATCGGAACATGTCCTGTAGTGGGGGATAGCCCGGCGAAAGCCGGATTAATACCGCATACGATCCATGGATGAAAGCGGGGGACCTTCGGGCCTCGCGCTATAGGGTTGGCCGATGGCTGATTAGCTAGTTGGTGGGGTAAAGGCCTACCAAGGCGACGATCAGTAGCTGGTCTGAGAGGACGACCAGCCACACTGGGACTGAGACACGGCCCAGACTCCTACGGGAGGCAGCAGTGGGGAATTTTGGACAATGGGCGAAAGCCTGATCCAGCAATGCCGCGTGTGTGAAGAAGGCCTTCGGGTTGTAAAGCACTTTTGTCCGGAAAGAAATCCTTGGCTCTAATACAGTCGGGGGATGACGGTACCGGAAGAATAAGCACCGGCTAACTACGTGCCAGCAGCCGCGGTAATACGTAGGGTGCAAGCGTTAATCGGAATTACTGGGCGTAAAGCGTGCGCAGGCGGTTTGCTAAGACCGATGTGAAATCCCCGGGCTCAACCTGGGAACTGCATTGGTGACTGGCAGGCTAGAGTATGGCAGAGGGGGGTAGAATTCCACGTGTAGCAGTGAAATGC

>SUR-180 AGTAACGCGTGGGAATCTACCTTTTGCTACGGAATAACGCAGGGAAACTTGTGCTAATACCGTATGTGTCCTTCGGGAGAAAGATTTATCGGCAAGAGATGAGCCCGCGTTGGATTAGCTAGTTGGTGGGGTAAAGGCCTACCAAGGCGACGATCCATAGCTGGTCTGAGAGGATGATCAGCCACATTGGGACTGAGACACGGCCCAAACTCCTACGGGAGGCAGCAGTGGGGAATATTGGACAATGGGCGCAAGCCTGATCCAGCCATGCCGCGTGAGTGATGAAGGCCCTAGGGTTGTAAAGCTCTTTCACCGGAGAAGATAATGACGGTATCCGGAGAAGAAGCCCCGGCTAACTTCGTGCCAGCAGCCGCGGTAATACGAAGGGGGCTAGCGTTGTTCGGAATTACTGGGCGTAAAGCGCACGTAGGCGGATCGATCAGTCAGGGGTGAAATCCCAGGGCTCAACCCTGGAACTGCCTTTGATACTGTCGATCTGGAGTATGGAAGAGGTGAGTGGAATTCCGAGTGTAGAGGTGAAATTCGTAGATATTCGGAGGAACACCAGTGGCGAAGGCGGCTCACTGGTCCATTACTGACGCTGAGGTGCGAAAGCGTGGGGAGC

>SUR-181 AAGCGCTGGAAACGGCGTCTAATACTGGATACGAACCGTGGAGGCATCTTCAACGGTTGGAAAGATTTTTTGGTCAGGGATGAGCTCGCGGCCTATCAGCTTGTTGGTGAGGTAATGGCTCACCAAGGCGTCGACGGGTAGCCGGCCTGAGAGGGTGACCGGCCACACTGGGACTGAGACACGGCCCAGACTCCTACGGGAGGCAGCAGTGGGGAATATTGCACAATGGGCGAAAGCCTGATGCAGCAACGCCGCGTGAGGGATGACGGCCTTCGGGTTGTAAACCTCTTTTAGCAGGGAAGAAGCGAAAGTGACGGTACCTGCAGAAAAAGCGCCGGCTAACTACGTGCCAGCAGCCGCGGTAATACGTAGGGCGCAAGCGTTATCCGGAATTATTGGGCGTAAAGAGCTCGTAGGCGGTTTGTCGCGTCTGCTGTGAAATCCCGAGGCTCAACTTCGGGTCTGCAGTGGGTACGGGCAGACTAGAGTGCGGTAGGGGAGATTGGAATTCCTGGTGTAGCGGTGGAATGCGCAGATATCAGGAGGAACACCGATGGCGAAGGCAGATCTCTGGGCCGT

>SUR-182 GGGGGACCTTCGGGCCTCTTGCCATCGGATGTGCCCAGATGGGATTAGCTATTAGGTGGGGTAATGGCTCACCTAGGCGACGATCCCTAGCTGGTCTGAGAGGATGACCAGCCACACTGGAACTGAGACACGGTCCAGACTCCTACGGGAGGCAGCAGTGGGGAATATTGCACAATGGGCGCAAGCCTGATGCATCCATGCCGCGTGTGTGAAGAAGGCCTTCGGGTTGTAAAGCACTTTCAGCGGGGAGGAAGGCAATACGGTTAATAACCGTGTTGATTGACGTTACCCGCATAAGAAGCACCGGCTAACTCCGTGCCAGCAGCCGCGGTAATACGGAGGGTGCAAGCGTTAATCGGAATTACTGGGCGTAAAGCGCACGCAGGCGGTCTGTCAAGTCGGATGTGAAATCCCCGGGCTCAACCTGGGAACTGCATTCGAAACTGGCAGGCTTGAGTCTTGTA

>SUR-183 CTGCCTGTAAGACTGGGATAACTCCGGGAAACCGGGGCTAATACCGGATGCTTGTTTGAACCGCATGGTTCAAACATAAAAAGTGGCTTTTCGCTACCACTTACAGATGGACCCGCGGCGCATTAGCTAGTTGGTGGGGTAACGGCTCACCAAGGCAACCATGCGTAGCCGACCTGAGAGGGTGATCGGCCACACTGGGACTGAAACACGGCCCAGACTCCTACGGGAGGCAGCATTAGGGAATCTTCCGCAATGGACGAAAGTCTGACGGAGCAACGCCGCGTGAGTGATGAAGGTTTTCGGATCGTAAAACTCTGTTGTTAGGGAAGAACAAGTACCGTTCAAACAGGGCGGTACCTTGACGGTACCTAACCAGAAAGCCACGGCTAACTACGTGCCAGCACCCGCGGTAATACGTAGGTGGCAAGCGTTGTCCGGAATTATTGGGCGTAA

>SUR-184 CCCCTGACTCTGGGATAAGCGCTGGAAACGGCGTCTAATACTGGATACGAACCGTGGAGGCATCTTCAACGGTTGGAAAGATTTTTTGGTCAGGGATGAGCTCGCGGCCTATCAGCTTGTTGGTGAGGTAATGGCTCACCAAGGCGTCGACGGGTAGCCGGCCTGAGAGGGTGACCGGCCACACTGGGACTGAGACACGGCCCAGACTCCTACGGGAGGCAGCAGTGGGGAATATTGCACAATGGGCGAAAGCCTGATGCAGCAACGCCGCGTGAGGGATGACGGCCTTCGGGTTGTAAACCTCTTTTAGCAGGGAAGAAGCGAAAGTGACGGTACCTGCAGAAAAAGCGCCGGCTAACTACGTGCCAGCAGCCGCGGTAATACGTAGGGCGCAAGCGTTATCCGGAATTATTGGGCGTAAAGAGCTCGTAGGCGGTTTGTCGCGTCTGCTGTGAAATCCCGAGGCTCAACTTCGGGTCTGCAGTGGGTACGGGCAGACTAGAGTGCGGTAGGGGAGATTGGAATTCCTGGTGTAGCGGTGGAATGCGCAGATATCAGGAGGAACACC

>SUR-216 ATAATGTCGCAAGACCAAAGAGGGGGACCTTCGGGCCTCTTGCCCTCAGATGTGCCCATATGGGATTATCTAATGTGTGGGGTAATGGCTCACCTAGGAGACTATCCCTATCTGGTCTGAGAGGATGACCACCCACTCTGGAACTGACACACGGTCCACACTCCTACGGGGGGCAGCAGTGGGGAATATTGCACAGTGGGCGCAAGCCTGATGCACCCATGCCGCGTGTGTGAAAAAGGCCTTCGGGTTGTAAAACTCTTTCAGCGGGGAAGAAGGCTATAGGGTTAATAACCGTGTTGATTGACTTTACCCGCAAAAAAAGCACCGGCTAACTCCGTGCCAGCACCCGCGGTAATACAGAGGGTGCAAGCGTTAATCAGAATTACTGGGCGTAAAGCGCACGCGCGCGGTCTGTCAAGTCAGATGTGAAATCCCCGGTCTCAACCTGGGAACTGCATTCAAAACTGGCGGGCTTGAGTCTTGTAGAGGGGGGT

>SUR-220 GGTGAGTAATGTCTGGGAAACTGCCCGATGGAGGGGGATAACTACTGGAAACGGTAGCTAATACCGCATAACGTCGCAAGACCAAAGAGGGGGACCTTCGGGCCTCTTGCCATCGGATGTGCCCAGATGGGATTAGCTAGTAGGTGGGGTAATGGCTCACCTAGGCGACGATCCCTAGCTGGTCTGAGAGGATGACCAGCCACACTGGAACTGAGACACGGTCCAGACTCCTACGGGAGGCAGCAGTGGGGAATATTGCACAATGGGCGCAAGCCTGATGCAGCCATGCCGCGTGTGTGAAGAAGGCCTTCGGGTTGTAAAGCACTTTCAGCGGGGAGGAAGGCAATACGGTTAATAACCGTGTTGATTGACGTTACCCGCAGAAGAAGCACCGGCTAACTCCGTGCCAGCAGCCGCGGTAATACGGAGGGTGCAAGCGTTAATCGGAATTACTGGGCGTAAAGCGCACGCAGGCGGTCTGTCAAGTCGGATGTGAAATCCCCGGGCTCAACCTGGGAACTGCATTCGAAACTGGCAGGCTTGAGTCTTGTAGAGGGGGGTAGAATTCCAGGTGTAGCGGTGAAATGCGTAGAGATCTGG

>SUR-240 GGAATAACAGTTAGAAATGACTGCTAATACCGGATGATGTCTTCGGACCAAAGATTTATCGGCAAGAGATGAGCCCGCGTAGGATTAGGTAGTTGGTGGGGTAAAGGCCTACCAAGCCGACGATCCTTAGCTGGTCTGAGAGGATGATCAGCCACACTGGGACTGAGACACGGCCCAGACTCCTACGGGAGGCAGCAGTGGGGAATATTGGACAATGGGGGCAACCCTGATCCAGCAATGCCGCGTGAGTGATGAAGGCCTTAGGGTTGTAAAGCTCTTTTACCAGGGATGATAATGACAGTACCTGGAGAATAAGCTCCGGCTAACTCCGTGCCAGCAGCCGCGGTAATACGGAGGGAGCTAGCGTTGTTCGGAATTACTGGGCGTAAAGCGCACGTAGGCGGTTACTCAAGTCAGAGGTGAAAGCCCGGGGCTCAACCCCGGAACTGCCTTTGAAACTAGGTAACTAGAATCCTGGAGAGGTGAGTGGAATTCCGAGTGTAGAGGTGAAATTCGTAGATATTCGGAAGAACACCAGTGGCGAAGGCGGCTCACTGGACAGGTATTGACGCTGAGGTGCGAAAGCGTGGGGAGCAAACAGGATTAGATACCCTGGTAGTCCACGCCGTAAACGA

>SUR-270 CGTGGGAACCTGCCCTTTGGTTCGGAATAACTCCGGGAAACTGGAGCTAATACCGGATGAGCCCTGTGGTTGTGGAGATCATAGGGGAAAGATTTATCGCCGAAGGAGGGGCCCGCGTCCGATTAGGTAGTTGGTGAGGTAACGGCTCACCAAGCCGACGATCGGTAGCTGGTCTGAGAGGATGATCAGCCACACTGGGACTGAGACACGGCCCAGACTCCTACGGGAGGCAGCAGTGGGGAATATTGGACAATGGGCGCAAGCCTGATCCAGCAATGCCGCGTGAGTGATGAAGGCCTTAGGGTTGTAAAGCTCTTTCGCACGTGACGATGATGACGGTAACGTGAGAAGAAGCCCCGGCTAACTTCGTGCCAGCAGCCGCGGTAATACGAAGGGGGCTAGCGTTGTTCGGAATTACTGGGCGTAAAGGGCGCGTAGGCGGCTGTTCAAGTCAGGCGTGAAATCCCCGGGCTTAACCTGGGAATTGCGTTTGAGACTGAGCGGCTTGAGTTCGGGAGAGGTGAGTGGAATTCCCAGTGTAGAGGTGAAATTCGTAGATATTGGGAAGAACAC

>SUR-283 AAACTGCCCGATGGAGGGGGATAACTACTGGAAACGGTACCTAATACCGCATAATGTCGCAAGACCAAAGAGGGGGACCTTCGGGCCTCTTGCCATCGGATGTGCCCAGATGGGATTAGCTTGTTGGTGAGGTAATGGCTCACCAAGGCGACGATCCCTAGCTGGTCTGAGAGGATGACCAGCCACACTGGAACTGAGACACGGTCCAGACTCCTACGGGAGGCACCAGTGGGGAATATTGCACAATGGGCGCAAGCCTGATGCAGCCATGCCGCGTGTGTGAAAAAGGCCTTCGGGTTGTAAAGTACTTTCAGCGGGGAGGAAGGCGATACGGTTAATAACCGTGTTGATTGACGTTACCCGCAGAAAAAGCACCGGCTAACTCCGTGCCAGCAGCCGCGGTAATACGGAGGGTGCAAGCGTTAATCGGAATTACTGGGCGTAAAGCGCACGCAGGCGGTCTGTCAAGTCGGATGTGAAATCCCCG

>SUR-284 GGGTGAGTAATGTCTGGGAAACTGCCCGATGGAGGGGGATAACTACTGGAAACGGTAGCTAATACCGCATAACGTCGCAAGACCAAAGAGGGGGACCTTCGGGCCTCTTGCCATCGGATGTGCCCAGATGGGATTAGCTAGTAGGTGGGGTAATGGCTCACCTAGGCGACGATCCCTAGCTGGTCTGAGAGGATGACCAGCCACACTGGAACTGAGACACGGTCCAGACTCCTACGGGAGGCAGCAGTGGGGAATATTGCACAATGGGCGCAAGCCTGATGCAGCCATGCCGCGTGTGTGAAGAAGGCCTTCGGGTTGTAAAGCACTTTCAGCGGGGAGGAAGGCAATACGGTTAATAACCGTGTTGATTGACGTTACCCGCAGAAGAAGCACCGGCTAACTCCGTGCCAGCAGCCGCGGTAATACGGAGGGTGCAAGCGTTAATCGGAATTACTGGGCGTAAAGCGCACGCAGGCGGTCTGTCAAGTCGGATGTGAAATCCCCGGGCTCAACCTGGGAACTGCATTCGAAACTGGCAGGCTTGAGTCTTGTAGAGGGGGGTAGAATTC

>SUR-286 AATGTCTGGGAAACTGCCCGATGGAGGGGGATAACTACTGGAAACGGTAGCTAATACCGCATAATGTCGCAAGACCAAAGAGGGGGACCTTCGGGCCTCTTGCCATCGGATGTGCCCAGATGGGATTAGCTTGTTGGTGAGGTAATGGCTCACCAAGGCGACGATCCCTAGCTGGTCTGAGAGGATGACCAGCCACACTGGAACTGAGACACGGTCCAGACTCCTACGGGAGGCAGCAGTGGGGAATATTGCACAATGGGCGCAAGCCTGATGCAGCCATGCCGCGTGTGTGAAGAAGGCCTTCGGGTTGTAAAGTACTTTCAGCGGGGAGGAAGGCAATACGGTTAATAACCGTGTTGATTGACGTTACCCGCAGAAGAAGCACCGGCTAACTCCGTGCCAGCAGCCGCGGTAATACGGAGGGTGCAAGCGTTAATCGGAATTACTGGGCGTAAAGCGCACGCAGGCGGTCTGTCAAGTCGGATGTGAAATCCCCGGGCTCAACCTGGGAACTGCATTCGAAACTGGCAGGCTTGAGTCTTGTA

>SUR-288 GGCGGACGGGTGAGTAATGTCTGGGAAACTGCCCGATGGAGGGGGATAACTACTGGAAACGGTAGCTAATACCGCATAATGTCGCAAGACCAAAGAGGGGGACCTTCGGGCCTCTTGCCATCGGATGTGCCCAGATGGGATTAGCTTGTTGGTGAGGTAATGGCTCACCAAGGCGACGATCCCTAGCTGGTCTGAGAGGATGACCAGCCACACTGGAACTGAGACACGGTCCACACTCCTACGGGAGGCAGCAGTGGGGAATATTGCACAATGGGCGCAAGCCTGATGCACCCATGCCGCGTGTGTGAAGAAGGCCTTCGGGTTGTAAAGTACTTTCAGCGGGGAGGAAGGCAATACGGTTAATAACCGTGTTGATTGACGTTACCCGCACAAAAAGCACCGGCTAACTCCGTGCCAGCAGCCGCGGTAATACGGAGGGTGCAAGCGTTAATCGGAATTACTGGGCGTAAAGCGCACGCAGGCGGTCTGTCAAGTCGGATGTGAAATCCCCGGGCTCAACCTGGGAACTGCATTCGAAACT

>SUR-291 GCTTCGGAATAACAGTTAAAAATGACTGCTAATACCGGATGATGTCTTCGGACCAAAGATTTATCGGCAAGAGATGAGCCCGCGTAGGATTAGGTAGTTGGTGGGGTAAAGGCCTACCAAGCCGACGATCCTTAGCTGGTCTGAGAGGATGATCAGCCACACTGGGACTGAGACACGGCCCAGACTCCTACGGGAGGCAGCAGTGGGGAATATTGGACAATGGGGGCAACCCTGATCCAGCAATGCCGCGTGAGTGATGAAGGCCTTAGGGTTGTAAAGCTCTTTTACCAGGGATGATAATGACAGTACCTGGAGAATAAGCTCCGGCTAACTCCGTGCCAGCAGCCGCGGTAATACGGAGGGAGCTAGCGTTGTTCGGAATTACTGGGCGTAAAGCGCACGTAGGCGGTTACTCAAGTCAGAGGTGAAAGCCCGGGGCTCAACCCCGGAACTGCCTTTGAAACTAGGTAACTAGAATCCTGG

>SUR-302 GCTCCTGATGGCGAGTGGCGAACGGGTGAGTAATATATCGGAACGTGCCCTAGAGTGGGGGATAACTAGTCGAAAGATTAGCTAATACCGCATACGATCTACGGATGAAAGTGGGGGATCGCAAGACCTCATGCTCCTGGAGCGGCCGATATCTGATTAGCTAGTTGGTGAGGTAAAAGCTCACCAAGGCGACGATCAGTAGCTGGTCTGAGAGGACGACCAGCCACACTGGGACTGAGACACGGCCCAGACTCCTACGGGAGGCAGCAGTGGGGAATTTTGGACAATGGGGGCAACCCTGATCCAGCAATGCCGCGTGAGTGAAGAAGGCCTTCGGGTTGTAAAGCTCTTTTGTCAGGGAAGAAACGGTTTTGGCTAATATCCAGAACTAATGACGGTACCTGAAGAATAAGCACCGGCTAACTACGTGCCAGCAGCCGCGGTAATACGTAGGGTGCAAGCGTTAATCGGAATTACTGGGCGTAAAGCGTGCGCAGGCGGTTGTGTAAGACAGATGTGAAATCCCCGGGCTCAACCTGGGAATTGCATTTGTGACTGCACGGCTAGAGTGTGTCAGAGGGGGGTAGAATTCCACGTGTAGCAGTGAAATGCGTAGATATGTGGAGGAATACCGATGG

>SUR-304 TGACGAGTGGCGGACGGGTGAGTAATGTCTGGGAAACTGCCCGATGGAGGGGGATAACTACTGGAAACGGTAGCTAATACCGCATAATGTCGCAAGACCAAAGAGGGGGACCTTCGGGCCTCTTGCCATCGGATGTGCCCAGATGGGATTAGCTTGTTGGTGAGGTAATGGCTCACCAAGGCGACGATCCCTAGCTGGTCTGAGAGGATGACCAGCCACACTGGAACTGAGACACGGTCCAGACTCCTACGGGAGGCAGCAGTGGGGAATATTGCACAATGGGCGCAAGCCTGATGCAGCCATGCCGCGTGTGTGAAGAAGGCCTTCGGGTTGTAAAGTACTTTCAGCGGGGAGGAAGGCAATACGGTTAATAACCGTGTTGATTGACGTTACCCGCAGAAGAAGCACCGGCTAACTCCGTGCCAGCAGCCGCGGTAATACGGAGGGTGCAAGCGTTAATCGGAATTACTGGGCGTAAAGCGCACGCAGGCGGTCTGTCAAGTCGGATGTGAAATCCCCGGGCTCAACCTGGGAACTGCATTCGAAACTGGCAGGCTTGAGTCTTGTAGAGGGGGGTAGAATTCCAGGTGTAGCGGTGAAATGCGTAGAGATCTGGAGGAA

>SUR-309 TGACGAGTGGCGAACGGGTGAGTAATGTATCGGAACGTGCCCAGTCGTGGGGGATAACTGCTCGAAAGAGCAGCTAATACCGCATACGACCTGAGGGTGAAAGCGGGGGATCGCAAGACCTCGCGCGATTGGAGCGGCCGATATCAGATTAGGTAGTTGGTGGGGTAAAGGCCTACCAAGCCGACGATCTGTAGCTGGTCTGAGAGGACGACCAGCCACACTGGGACTGAGACACGGCCCAGACTCCTACGGGAGGCAGCAGTGGGGAATTTTGGACAATGGGCGCAAGCCTGATCCAGCCATGCCGCGTGCGGGAAGAAGGCCTTCGGGTTGTAAACCGCTTTTGTCAGGGAAGAAAAGCCTTTGGTTAATACCTAGAGGTGATGACGGTACCTGAAGAATAAGCACCGGCTAACTACGTGCCAGCAGCCGCGGTAATACGTAGGGTGCAAGCGTTAATCGGAATTACTGGGCGTAAAGCGTGCGCAGGCGGTGATGCAAGACAGAGGTGAAATCCCCGGGCTCAACCTGGGAACTGCCTTTGTGACTGCATGACTAGAGTACGGTAGAGGGGGATGGAATTCCGCGTGTAGCAGTGAAATGCGTAGATATGCGGAGGAACACCGATGGCGAAGGCAATCCCCTGGACCTGTACTGACGCTCATGCACGAAAGCGTGGGGAGCAAACAGGATTAGATACCCTGGTAGTCCACGCCCTAAAC

>SUR-310 TAACGCGTGGGAATCTACCCAACCCTACGGAATAACGCATGGAAACGTGTGCTAATACCGTATACGCCCTACGGGGGAAAGATTTATCGGGGATGGATGAGCCCGCGTTGGATTAGCTAGTTGGTGGGGTAAAGGCCTACCAAGGCGACGATCCATAGCTGGTCTGAGAGGATGATCAGCCACATTGGGACTGAGACACGGCCCAAACTCCTACGGGAGGCAGCAGTGGGGAATATTGGACAATGGGCGCAAGCCTGATCCAGCCATGCCGCGTGAGTGATGAAGGCCTTAGGGTTGTAAAGCTCTTTCAACGGTGAAGATAATGACGGTAACCGTAGAAGAAGCCCCGGCTAACTTCGTGCCAGCAGCCGCGGTAATACGAAGGGGGCTAGCGTTGTTCGGAATTACTGGGCGTAAAGCGCACGTAGGCGGACTTTTAAGTCAGGGGTGAAATCCCAGGGCTCAACCCTGGAACTGCCTTTGATACTGGAAGTCTGGAGTATGGAAGAGGTGAGTGGAATTCCGAGTGTAGAGGTGAAATTCGTAGATATTCGGAGGAACACCAGTGGCGAAGGCGGCTCACTGGTCCATTACTGACGCTGAGGTGCGAAAGCGTGGGGAGCA

>SUR-311 ACGAGTGGCGGACGGGTGAGTAATGTCTGGGAAACTGCCCGATGGAGGGGGATAACTACTGGAAACGGTAGCTAATACCGCATAACGTCGCAAGACCAAAGAGGGGGACCTTCGGGCCTCTTGCCATCGGATGTGCCCAGATGGGATTAGCTAGTAGGTGGGGTAATGGCTCACCTAGGCGACGATCCCTAGCTGGTCTGAGAGGATGACCAGCCACACTGGAACTGAGACACGGTCCAGACTCCTACGGGAGGCAGCAGTGGGGAATATTGCACAATGGGCGCAAGCCTGATGCAGCCATGCCGCGTGTGTGAAGAAGGCCTTCGGGTTGTAAAGCACTTTCAGCGGGGAGGAAGGCAATACGGTTAATAACCGTGTTGATTGACGTTACCCGCAGAAGAAGCACCGGCTAACTCCGTGCCAGCAGCCGCGGTAATACGGAGGGTGCAAGCGTTAATCGGAATTACTGGGCGTAAAGCGCACGCAGGCGGTCTGTCAAGTCGGATGTGAAATCCCCGGGCTCAACCTGGGAACTGCATTCGAAACTGGCAGGCTTGAGTCTTGTAGAGGGGGGTAGAATTCCAGGTGTAGCGGTGAAATGCGTAGAGATCTGGAGGAATACCGGTGGCGAAGGCG

>SUR-315 TCGGGTGACGAGTGGCGGACGGGTGAGTAATGTCTGGGAAACTGCCCGATGGAGGGGGATAACTACTGGAAACGGTAGCTAATACCGCATAACGTCGCAAGACCAAAGAGGGGGACCTTCGGGCCTCTTGCCATCGGATGTGCCCAGATGGGATTAGCTAGTAGGTGGGGTAATGGCTCACCTAGGCGACGATCCCTAGCTGGTCTGAGAGGATGACCAGCCACACTGGAACTGAGACACGGTCCAGACTCCTACGGGAGGCAGCAGTGGGGAATATTGCACAATGGGCGCAAGCCTGATGCAGCCATGCCGCGTGTGTGAAGAAGGCCTTCGGGTTGTAAAGCACTTTCAGCGGGGAGGAAGGCAATACGGTTAATAACCGTGTTGATTGACGTTACCCGCAGAAGAAGCACCGGCTAACTCCGTGCCAGCAGCCGCGGTAATACGGAGGGTGCAAGCGTTAATCGGAATTACTGGGCGTAAAGCGCACGCAGGCGGTCTGTCAAGTCGGATGTGAAATCCCCGGGCTCAACCTGGGAACTGCATTCGAAACTGGCAGGCTTGAGTCTTGTAGAGGGGGGTAGAATTCCAGGTGTAGCGGTGAAATGCGTAGAGATCTGGAGGAATACCGGTGGCGAAGGCGGCCCCCTGGACAAA

>SUR-316 AGGGGGACCTTCTGGCCTCTTGCCGTCTGATGTGCCCAGATGGGATTAGCTAGTTGTGGGGTAATGGCTCACCTAGGCGACGATCCCTAGCTGGTCTGAGAGGATGACCACCCACACTGAAACTGAGACACGGTCCACACTCCTACGGGAGGCAGCAGTGGGGAATATTGCACAATGGGCGCAAGCCTGATGCAGCCATGCCGCGTGTGTGACGAAGGCCTTCGGGTTGTAAAGCACTTTCAGCGGGGAGGAAGGCAATACGGTTAATAACCGTGTTGATTGACCTTACCCGCAGAAGAAGCACCGGCTAACTCCGTGCCAGCAGCCGCGGTAATACGGAGGGGGCAAGCGTTAATCCGAAT

>SUR-317 TGGAGGGGGATAACTACTGGAAACGGTAGCTAATACCGCATAACGTCGCAAGACCAAAGAGGGGGACCTTCGGGCCTCTTGCCATCGGATGTGCCCAGATGGGATTAGCTAGTAGGTGGGGTAATGGCTCACCTAGGCGACGATCCCTAGCTGGTCTGAGAGGATGACCAGCCACACTGGAACTGAGACACGGTCCAGACTCCTACGGGAGGCAGCAGTGGGGAATATTGCACAATGGGCGCAAGCCTGATGCAGCCATGCCGCGTGTGTGAAGAAGGCCTTCGGGTTGTAAAGCACTTTCAGCGGGGAGGAAGGCAATACGGTTAATAACCGTGTTGATTGACGTTACCCGCAGAAGAAGCACCGGCTAACTCCGTGCCAGCAGCCGCGGTAATACGGAGGGTGCAAGCGTTAATCGGAATTACTGGGCGTAAAGCGCACGCAGGCGGTCTGTCAAGTCGGATGTGAAATCCCCGGGCTCAACCTGGGAACTGCATTCGAAACTGGCAGGCTTGAGTCTTGTAGAGGGGGGTAGAATTCCAGGTGTAGCGGTGAAATGCG

>SUR-318 ACTGCCCGATGGAGGGGGATAACTACTGGAAACGGTAGCTAATACCGCATAATGTCGCAAGACCAAAGAGGGGGACCTTCGGGCCTCTTGCCATCGGATGTGCCCAGATGGGATTAGCTTGTTGGTGAGGTAATGGCTCACCAAGGCGACGATCCCTAGCTGGTCTGAGAGGATGACCAGCCACACTGGAACTGAGACACGGTCCACACTCCTACGGGAGGCAGCAGTGGGGAATATTGCACAATGGGCGCAAGCCTGATGCAGCCATGCCGCGTGTGTGAAGAAGGCCTTCGGGTTGTAAAGTACTTTCAGCGGGGAGGAAGGCAATACGGTTAATAACCGTGTTGATTGACGTTACCCGCAGAAGAAGCACCGGCTAACTCCGTGCCAGCAGCCGCGGTAATACGGAGGGTGCAAGCGTTAATCGGAATTACTGGGCGTAAAGCGCACGCAGGCGGTCTGTCAAGTCGGATGTGAAATCCCCGGGCTCAACCTGGGAACTGCATTCGAAACTGGCAGGCTTGAGTCTTGTAGAGGGGGGTAGAATTCCAGGTGTAGCGGTGAAATGC
